# Single-Particle Entanglement Dynamics in Complex Systems

**DOI:** 10.3390/e28010029

**Published:** 2025-12-25

**Authors:** Devanshu Shekhar, Pragya Shukla

**Affiliations:** Department of Physics, Indian Institute of Technology, Kharagpur 721302, West Bengal, India; devanshu2096@gmail.com

**Keywords:** entanglement, dynamics, Anderson model, Rosenzweig–Porter ensemble, random matrix theory

## Abstract

We analyze the effect of varying system conditions on the single-particle entanglement entropy for an arbitrary eigenstate of a complex system that can be described by a multiparametric Gaussian ensemble. Our theoretical analysis leads to the identification of a single functional of the system parameters that governs the entropy dynamics. This reveals a sensitivity of the entropy to collective information content, characterized by the functional, instead of the individual system details. The functional can further be used to identify the universality classes as well as a deep web of connection underlying different quantum states.

## 1. Introduction

The ubiquitous complexity in real systems highlights the concept of entropy as an important tool for understanding behavior in diverse fields, e.g., classical thermodynamics, statistical physics, and information theory. Indeed, with its basic definition based on the randomness and uncertainty in the system, an underlying connection among its various forms, e.g., thermal, statistical, and information, is intuitively expected. This is also corroborated by many recent studies; e.g., the study [1] indicates that a consideration of the relationship between thermal and entanglement entropy can place significant constraints on the ground state entanglement structure for “natural” systems. Another study [2] indicates that the entanglement entropy of a topologically ordered system measures the maximal quasiparticle fluctuations and, as a consequence, corresponds to the thermal entropy of the quasiparticles at infinite temperature on the entanglement boundary. The potential connection with thermal entropy motivates us to analyze an important form of the entanglement entropy, namely ‘single-particle entanglement entropy’ (SPEE), developed to treat the ubiquitous indistinguishable particles.

The standard tools of entanglement rely on the tensor product structure of the state space of a composite quantum system. While such an underlying structure is always present in the case of distinguishable particles, the constraints on the particle statistics in the case of indistinguishable ones confine the available Hilbert space, thus rendering the standard approach inapplicable. To overcome this issue, an alternative tool was proposed in the past: it is based on an isomorphism (nonunique) of the Fock space to the state space of a composite (many modes) quantum system [3]. The idea in turn motivated the concept of single-particle entanglement (SPE): consider a particle confined on a bipartitioned lattice of arbitrary dimensions. The single-particle state of the lattice Hamiltonian can then be expressed as a tensor product of the site occupation number basis states in the second-quantized Fock space. The site entanglement in the two parts of the lattice can then be utilized as a criterion to analyze the aspects of single-particle wave dynamics where traditional criteria such as the inverse participation ratio, etc., are inadequate. Some examples in this context are wave dynamics under anisotropic system conditions, localization-to-delocalization phase transition, etc. Indeed, application of the entanglement statistics for characterization of the phase transitions has been studied in many previous studies [4,5,6,7,8,9], but most of them have been confined to bipartite or multipartite entanglement measures. Comparatively, less attention has been paid to single-particle entanglement analysis [10,11], which, albeit counterintuitive, has been proven to be a genuine resource in quantum information processing [3,12]. In this work, we pursue the analysis further by developing a common mathematical formulation of single-particle entanglement entropy dynamics for a wide range of Hamiltonians; the results are numerically verified for two prototypical Hamiltonians, viz., the three-dimensional (3D) Anderson model, representing a particle moving in a random potential with its hopping confined to some nearest neighbors, and the Rosenzweig–Porter ensemble, representing long-range interactions in the basis space.

The first and foremost requirement for the entanglement analysis of a physical system is the knowledge of its Hamiltonian in the physically motivated basis and thereafter its eigenstates. A realistic system is, however, almost always many-body, consisting of complicated interactions among its subunits. Even if the latter are well-known, e.g., Coulomb interactions, an exact determination of the matrix representation of the Hamiltonian is often not possible; this could occur, for example, due to technical issues involved in determination of the matrix elements (e.g., calculation of the integrals either by a theoretical or numerical route). Incomplete knowledge or error in their determination manifests itself by randomization of the matrix elements, which can, however, be of different types. For example, if a matrix element can be determined up to its average value and variance, the maximum entropy hypothesis predicts its distribution to be Gaussian. In case information is available about higher-order moments, it can lead to non-Gaussian distributions. Similarly, the existence of conservation laws can result in correlated distributions or other constraints on the matrix elements, e.g., column/row sum rule. In addition, not all elements need be random; some of them can be exactly determined. For example, for the Hamiltonian of a tight-binding lattice with on-site disorder and nearest neighbour non-random hopping, the diagonals in the site basis are randomly distributed but the off-diagonals are all non-random (given by the hopping strength). Indeed, the nature and type of the distribution of the matrix elements is sensitive to various system conditions, e.g., symmetry and conservation laws, dimensionality and boundary conditions, disorder, etc., and can vary from one element to another. As a consequence, the Hamiltonian matrix is best represented by a system-dependent random matrix, with some or all elements randomly distributed. The information about the system appears through the distribution parameters of the matrix elements. As expected, the randomness underlying the matrix elements of the Hamiltonian also manifests in its eigenstates, and the distribution of the latter can be derived, in principle, from a multivariable integration over the Hamiltonian ensemble density (i.e., the JPDF of the matrix elements); this is achieved by a transformation of variables from the matrix space to the eigenvalue–eigenstate space and thereafter an integration over all eigenvalues and all other eigenfunctions except one of them. The information in turn leads to statistics of the entanglement measures of the remaining eigenstate.

A multivariable integration of the ensemble density of the Hamiltonian is in general technically complicated. In addition, the interactions among various subunits of a many-body system are in general sensitive to a host of system conditions, both of static as well as dynamic type. A variation in these conditions can lead to changes in the mutual interactions among subunits and thereby the ensemble density. This in turn is expected to manifest in the behavior of a typical many-body state and thereby entanglement measures and can lead to serious technical issues: even if the integration is achieved by some approximations, these are often specific to system parameters and may not be applicable once the parameters are changed. The integration route is therefore not often a viable option and motivates a search for alternative tools that take into account arbitrary variation in the system parameters.

The search for an alternative tool to determine quantum correlations for a general class of Hamiltonians represented by multiparametric Gaussian ensembles [13,14,15] motivates us to seek a common mathematical formulation for the single-particle entanglement statistics. As discussed in [15], the joint probability distribution function (JPDF) of the components of an arbitrary eigenstate (referred to as the state JPDF hereafter for brevity) for Hermitian operators represented by multivariate Gaussian ensembles undergoes a multiparametric evolution as the ensemble parameters vary. For a specific variation in the latter, the evolution describes a diffusion in state space and can be exactly derived from the ensemble density. The diffusion equation has an additional benefit: it provides a common mathematical formulation, referred as the complexity parameter formulation, of the state JPDF for a wide range of Hamiltonians where the system information is contained in a single functional of all system parameters. We apply the above formulation in the present work and thereby derive the evolution equation for the single-particle entanglement measures and their solutions.

We proceed as follows. We begin in Section 2 by introducing the standard definitions of the single-particle entanglement entropy (SPEE). This is followed by Section 3, describing two prototypical examples of a large class of single-particle Hamiltonians for which our formulation is intended. Section 4 revisits the complexity parameter formulation, briefly explaining how the changing system conditions lead to an evolution of the JPDF of the matrix elements and in turn of the eigenfunction components. The JPDF dynamics of the eigenfunction components is later used to obtain the evolution equation of the single-particle entanglement in Section 5. Finally, in Section 6, we present the exact diagonalization results of the examples introduced in Section 3 as a validation of our theoretical claims and conclude in Section 7 with a summary of our results and future directions. The theoretical derivations of our results involve detailed technical background material, already published several times; to keep this paper self-contained but to avoid its defocusing, we have moved these details to the Supplementary Material. For the reader’s convenience, we also include a brief description of the symbols used in this paper in Appendix A.

## 2. Single-Particle Entanglement Measure

**Figure 1:**
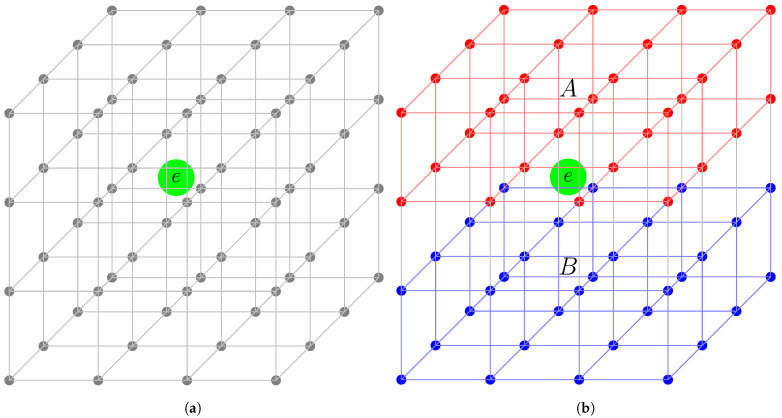
(**a**) An electron in a 3D lattice. (**b**) Horizontal bipartitions of the lattice into two subparts A and B.

Consider the dynamics of a single particle in a lattice of arbitrary dimension *D*, bipartitioned into parts *A* and *B*, created, e.g., by slicing the 3D lattice horizontally, vertically, or in an arbitrary direction as per requirement (as illustrated in Figure 1 by a schematic diagram). With each subpart associated with an occupation probability of the particle, it can be described by state |1〉 (subpart occupied) or |0〉 (subpart empty). An arbitrary state of the particle can then be written as a product over the occupation probabilities in the two parts. Based on whether the particle occupies the sites in subsystem A or B, two such product states are possible. An eigenstate of the Hamiltonian *H* can then be written as a superposition of the product states,

(1) $$|\psi \rangle ={|1\rangle }_{A}{|0\rangle }_{B}+{|0\rangle }_{A}{|1\rangle }_{B},$$

But as the probability $P_{A}$ or $P_{B}$ of the particle lying in subpart *A* or *B* depends on the number of sites therein, this implies ${|1\rangle }_{A}=\sum_{r\in A}\psi_{r}{|1\rangle }_{r}{⊗}_{r^{\prime }\neq r}{|0\rangle }_{r^{\prime }}$ and ${|0\rangle }_{A}={⊗}_{r\in A}{|0\rangle }_{r}$ and similarly for *B*. The state |ψ〉 can then be written as

(2) $$|\psi \rangle =\sum_{r\in A\cup B}\psi_{r}{|1\rangle }_{r}{⊗}_{r^{\prime }\neq r}{|0\rangle }_{r^{\prime }},$$

where ${|1\rangle }_{r}$ and ${|0\rangle }_{r}$ correspond to the occupation or vacancy of site *r*, respectively.

The formulation for the standard Von Neumann entropy of a pure quantum state is analogous to the one for the Shannon entropy. Based on similar ideas, the single-particle entanglement entropy (SPEE) is defined in terms of the probability of occupation of the subparts. Let $P_{A}$ be the probability of the particle occurring in part *A*,

(3) $$P_{A}\equiv {\langle 1|1\rangle }_{A}=\sum_{r\in A}{\left|\psi_{r}\right|}^{2},$$

The SPEE can then be defined as

(4) $$S_{A}\left(\rho_{A}\right)=-P_{A}\log P_{A}-\left(1-P_{A}\right)\log\left(1-P_{A}\right)=-P_{A}\log P_{A}-P_{B}\log P_{B}.$$

where $P_{B}\equiv 1-P_{A}$. In the ergodic limit, for a balanced bipartition implying equal occupancy of both subparts, we have $P_{A}=P_{B}\to 1/2$ and thereby $S_{A}\to \log\left(2\right)$. The other extreme limit corresponds to the particle being localized in one subpart, thereby implying $P_{A}=1,P_{B}=0$ or $P_{A}=0,P_{B}=1$; both these possibilities correspond to $S_{A}\to 0$. Consequently, $S_{A}$ varies between 0 and log(2) as the particle wavefunction starts extending from one subpart to another.

## 3. Hamiltonian and Ensemble Density

Equation (4) gives the SPEE for an eigenfunction of a single-particle Hamiltonian. However, for cases where the Hamiltonian is best described by an ensemble, it is imperative to consider the distribution of the eigenfunction over the ensemble and therefore SPEE statistics. The latter can be derived if the theoretical formulation for the ensemble density of Hamiltonians representing the particle is available. The natural query arising then is how to find the appropriate as well as mathematically tractable ensemble representing a given Hamiltonian. The next section elucidates our approach to construct the ensembles for two prototypical single-particle Hamiltonians; these are later used in the numerical analysis too.

(i) Anderson Hamiltonian: Consider a single electron moving in a random potential, e.g., a *D*-dimensional disordered lattice. Within tight-binding approximation, the dynamics can be described by the Hamiltonian [10,11,16,17]

(5) $$\begin{array}{c} H=\sum_{k}\epsilon_{k}\left|k\rangle \langle k\right|+\sum_{\langle k,l\rangle }t_{kl}|k\rangle \langle l|. \end{array}$$

where $t_{kl}=\langle k|H|l\rangle$ describes the tunneling amplitude of an electron between sites *k* and *l* and the summation $\sum_{\langle k,l\rangle }$ is over *z* neighboring sites. Based on the nature of the disorder, the ensemble representing *H* can be of various types. For example, for the independent Gaussian-distributed site energies $\epsilon_{k}$ with variance $v_{kk}=\sigma^{2}$, the probability density of $H_{kk}$ can be written as $\rho_{kk}\left(H_{kk}\right)=e^{-{\left(H_{kk}-\epsilon_{k}\right)}^{2}/2\sigma^{2}}$. The hopping can be chosen to be isotropic or anisotropic and non-random or random (Gaussian). In the case of non-random, anisotropic hopping $b_{kl}$ between nearest neighbours, we have $\rho_{kl}\left(H_{kl}\right)=\delta \left(H_{kl}-b_{kl}\right)$ if k,l form nearest neighbour pairs and $\rho_{kl}\left(H_{kl}\right)=\delta \left(H_{kl}\right)$ if k,l are not nearest neighbours. The probability density $\rho \left(H\right)\equiv \prod_{\langle k,l\rangle }\rho_{kl}\left(H_{kl}\right)$ of the ensemble can therefore be given as

(6) $$\begin{array}{c} \rho_{1}\left(H\right)=C\exp\left[-\sum_{k}\frac{{\left(H_{kk}-\epsilon_{k}\right)}^{2}}{2\sigma^{2}}\right]\prod_{\langle k,l\rangle }\delta \left(H_{kl}-b_{kl}\right)\prod_{k,l\neq n.n}\delta \left(H_{kl}\right) \end{array}$$

with *C* as a normalization constant; $\prod_{\langle k,l\rangle }$ denotes the product over connected sites only (depends upon the number of neighbors). Similarly, in the case of random nearest neighbor hopping, we have

(7) $$\begin{array}{c} \rho_{1}\left(H\right)=C\exp\left[-\sum_{k}\frac{{\left(H_{kk}-\epsilon_{k}\right)}^{2}}{2\sigma^{2}}\right]\exp\left[-\sum_{k,l=n.n.}\frac{{\left(H_{kl}-b_{kl}\right)}^{2}}{v_{kl}}\right]\prod_{k,l\neq n.n.}\delta \left(H_{kl}\right) \end{array}$$

Using the Gaussian limit of the delta function, i.e., $\delta \left(x\right)=\lim_{v\to 0}\frac{1}{\sqrt{2\pi v^{2}}}e^{-x^{2}/2v^{2}}$, Equation (6) and Equation (7) can both be written in the form of a multiparametric Gaussian ensemble,

(8) $$\begin{array}{c} \rho \left(H;v,b\right)=\lim_{v\to 0}C\exp\left[-\sum_{k}\frac{{\left(H_{kl}-\epsilon_{k}\right)}^{2}}{2\sigma^{2}}-\sum_{k,l;k\neq l}\frac{{\left(H_{kl}-b_{kl}\right)}^{2}}{v_{kl}}\right], \end{array}$$

where $b_{kl}=0$ for disconnected sites and the notation $\lim_{v\to 0}$ corresponds to the limit $v_{kl}\to 0$ for all k,l-pairs corresponding to the non-random hopping or for disconnected sites.

(ii) Rosenzweig–Porter Ensemble: The Rosenzweig–Porter ensemble (RPE), a prototypical model often used for spectral analysis of the localization → delocalization transition [16,18,19,20], is described by the probability density [16]

(9) $$\begin{array}{c} \rho_{2}\left(H\right)\propto \exp\left[-\frac{1}{2}\sum_{i=1}^{N}H_{ii}^{2}-\left(1+\mu \right)\sum_{i,j=1;i<j}^{N}{\left|H_{ij}\right|}^{2}\right], \end{array}$$

i.e., a Hamiltonian with normally distributed diagonal entries and off-diagonal entries with variance controlled by an external parameter μ:

(10) $$\langle \delta H_{ij}^{2}\rangle =\delta_{ij}+\frac{1-\delta_{ij}}{1+\mu }.$$

We note that a typical Hamiltonian taken from the above ensemble describes, e.g., a long-range isotropic hopping in a lattice with on-site disorder. As discussed in [16], a choice of $\mu =cN^{\alpha }$, with *c* and α as arbitrary parameters, reveals the rich dynamics of a typical wavefunction of the Hamiltonian; it undergoes a localization → extended non-ergodic → ergodic transition of wavefunction dynamics with changing α for a fixed *N* [16]. For α=1 and 2, the spectral statistics is known to be critical and different from both Poisson (localized limit) and Wigner–Dyson (ergodic limit). The eigenfunction statistics in the bulk of the spectrum for these α-values is known to be multifractal.

## 4. Complexity Parameter Formulation of the Hamiltonian Ensemble

Our primary focus in the present work is to study the entanglement statistics of a typical eigenstate of a complex system represented by the Hamiltonian *H* in the single-particle approximation. As the examples discussed in the previous section indicate, a wide range of Hamiltonians can be represented by an ensemble of *N*-dimensional real-symmetric matrices *H*, described by an ensemble density,

(11) $$\begin{array}{c} \rho \left(H;v,b\right)=C\exp\left[-\sum_{k\leq l}\frac{{\left(H_{kl}-b_{kl}\right)}^{2}}{v_{kl}}\right], \end{array}$$

with $v_{kl}$ and $b_{kl}$ as the arbitrary variance and mean value of the matrix element $H_{kl}$ and *C* as the normalization constant. As the limit $v_{kl}\to 0$ corresponds to a non-random $H_{kl}$ taking a value $b_{kl}$, the above density can model a wide range of ensembles, including sparse as well as banded ones. As mentioned in Section 1, for ρ(H) to be an appropriate representation of the Hamiltonian, the ensemble parameters are expected to be sensitive to the system conditions. Some examples of such system parameters are disorder, dimensionality, boundary and topological conditions, system size, etc. Indeed, the ensemble densities in Equations (8) and (9) correspond to special cases of Equation (11).

The ensemble density in Equation (11) is a function of N(N+1)/2 matrix elements $H_{kl}$ and N(N+1) ensemble parameters $v_{kl},b_{kl}$. A variation in system conditions affects various interactions among subunits of the system and changes not only $H_{kl}$ but also their uncertainties and thereby distribution parameters. This in turn leads to changes in ρ(H) both in the matrix space as well as in the ensemble parameter space; the location of each matrix of the ensemble in the matrix space is changed and thereby the moments $\langle H_{kl}^{n}\rangle$ are averaged over the shifted ensemble. The dynamics give rise to many natural queries, a few of which are mentioned below.

(i) Whether the dynamics of ρ(H) in the ensemble parameter space has an exact equivalent in the matrix space. This information is relevant for following reason: We assume that the system under consideration is well-described by the ensemble in Equation (11) at a given instant of time. A change in the system conditions can, however, affect the matrix elements. The question is whether the system can still be described by the same ensemble with time-evolved ensemble parameters? Indeed, from Equation (11), a variation in either the matrix elements or the ensemble parameters can change the ensemble density. For an evolving ensemble to continue representing the system, it is desirable that the evolution of ρ(H) in *H*-matrix space is exactly mimicked by that in (v,b) space, where v,b refer to the variance and mean matrices, consisting of $v_{kl}$ and $b_{kl}$, respectively.

(ii) Does the dynamics indeed depend on individual details of all ensemble parameters or only respond to their collective influence? This query arises from ample evidence in many areas of complex systems that complexity, irrespective of its origin, is sensitive to very few details or relevant system parameters, often resulting in universality of the physical properties.

(iii) Whether the new ensemble still retains its Gaussian form (requires specific conditions on $\langle H_{kl}^{n}\rangle$) and whether it be accessed from the initial ensemble, in the ensemble parameter space, just by varying the parameters $v_{kl}$ and $b_{kl}$. On intuitive grounds, this is expected to be achieved only for specific dynamics in the matrix space.

### 4.1. Diffusion Equation for ρ(H)

Indeed, as discussed in detail in [13] (also in Section SI of the Supplementary Material), a specific drift dynamics of ρ(H) in (v,b)-space, generated by an operator $T\equiv \sum_{k\leq l}\left[\frac{2x_{kl}}{{\tilde{g}}_{kl}}\frac{\partial }{\partial h_{kl}}-\gamma b_{kl}\frac{\partial }{\partial b_{kl}}\right]$, can exactly mimic a diffusive dynamics with a finite drift in *H*-matrix space: Tρ=Lρ, where $L\equiv \sum_{k,l}\frac{\partial }{\partial H_{kl}}\left[\frac{g_{kl}}{2}\frac{\partial }{\partial H_{kl}}+\gamma H_{kl}\right]$. Here $x_{kl}\equiv 1-\gamma {\tilde{g}}_{kl}v_{kl}$, ${\tilde{g}}_{kl}=2-\delta_{kl}$, and $g_{kl}=1+\delta_{kl}$ and γ is a non-zero but otherwise arbitrary constant giving the variance of the matrix elements at the end of the evolution [13,16].

The above equation describes a multiparametric flow of the matrix elements in a real-symmetric matrix space from an arbitrary initial condition, say $H_{0}$. As discussed in detail in [13,16], the dynamics of ρ(H;v,b) in (v,b)-space can be mapped to another *M*-dimensional parametric space $t\equiv \{t_{1},\ldots ,t_{M}\}$, in which ρ(H;t) undergoes a single parametric evolution if following set of conditions is satisfied:

(12) $$\begin{array}{c} \frac{\partial \rho }{\partial t_{1}}=L\rho ,\;\;\frac{\partial \rho }{\partial t_{\alpha }}=0\;\forall \alpha >1, \end{array}$$

As is clear from the above, $t_{1}$ is the evolution parameter and $t_{2},\ldots ,t_{M}$ are the constants of the evolution. To distinguish from the (v,b)-space, hereafter the *t*-space will be referred as the complexity parameter space; its dimensionality *M* depends on the number of original ensemble parameters v,b undergoing variation, and in general M≤N(N+1) [13].

The parameters $t_{1},\ldots ,t_{M}$ can be obtained by solving the characteristic set of equations

(13) $$\begin{array}{c} \frac{df_{1}}{f_{1}}=\frac{df_{2}}{f_{2}}=\ldots =\frac{df_{M}}{f_{M}}=\frac{dt_{\alpha }}{\delta_{\alpha 1}} \end{array}$$

with $f_{j}=\left(1/2\right)\log\left|x_{kl}\right|+c_{j}$ for $j=1\to M_{1}$ and $f_{j}=\log\left|b_{kl}\right|+C_{j}$ for $j>M_{1}$, with $M_{1}$ referring to the number of non-zero $x_{kl}$.

A particular solution of the above equation can be given as

(14) $$\begin{array}{ccc} t_{1} & = & \sum_{\mu =1}^{M}q_{\mu ;1}\log\left|f_{\mu }\right|+c_{0}, \end{array}$$

(15) $$\begin{array}{ccc} t_{\alpha } & = & \sum_{\mu }q_{\mu ;\alpha }\log\left|f_{\mu }\right|,\;\;\;\;\;\alpha >1 \end{array}$$

with $q_{\mu ;\alpha }$ as arbitrary constants subjected to the condition $\sum q_{\mu ;\alpha }=\delta_{\alpha 1}$. While many solutions satisfying the above condition are possible, the appropriate solution is the one that is also applicable to the initial ensemble (as the constants of evolution are same for the initial ensemble). Indeed, the choice of constants $q_{\mu ;1}$ in Equation (14) depends on the evolving ensemble parameters. For example, if one of the $f_{\mu }$ remains zero throughout the evolution and does not participate in the evolution, the corresponding coefficient $q_{\mu ;1}$ can be set to zero. For the case in which all $f_{\mu }$ remain non-zero throughout the evolution, we have M=N(N+1), and it is appropriate to choose $q_{\mu ;1}=1/N\left(N+1\right)$ (for all μ). The choice fulfills the condition $\sum_{\mu =1}^{M}q_{\mu ;1}=1$, and $t_{1}$ can be given as

(16) $$\begin{array}{c} t_{1}=-\frac{1}{N(N+1)\gamma }\log\left[\prod_{k\leq l}\left|g_{kl}-2\gamma v_{kl}\right|{\left|b_{kl}\right|}^{2}\right]+\mathrm{constant}. \end{array}$$

To distinguish it from $t_{2},\ldots ,t_{M}$, hereafter $t_{1}$ is symbolically referred to as *Y*. As *Y* acts as the evolution parameter while $t_{2},\ldots ,t_{M}$ are the constants of evolution, the former can appropriately be referred to as the ensemble *complexity parameter* and the latter as complexity constants.

Being a sum over logarithmic functions of $v_{kl}$ and $b_{kl}$, *Y* can be regarded as an average distribution parameter, i.e., an average uncertainty that governs the statistics as system conditions change. Another definition of *Y* follows from an alternative derivation discussed in [21], implying it to be the average time-scale associated with accuracy fluctuations due to changing system conditions.

The transformation from the space of ensemble parameters to complexity parameters implies the transformation of ρ(H;v,b) in Equation (11) to $\rho (Y,t_{2},\ldots t_{M})$ (the latter given by Equation (10) in Section SI of the Supplementary Material). The ensembles with different (v,b) sets can in general have different values of $t_{1},\ldots ,t_{M}$ and therefore are represented by different points in the complexity parameter space. The common mathematical framework given by Equation (12) for the wide range of ensembles represented by ρ(H) in Equation (11) suggests an analogous formulation for the eigenvalues and eigenfunctions and their classification into an infinite range of universality classes defined by a single parameter *Y* for a given set of the constants of evolution. The latter in turn depend on the matrix constraints, e.g., whether the *H*-matrix is subjected to the same symmetry or conservation laws. The ensembles with the same set of $t_{2},\ldots ,t_{M}$ (i.e., same type of matrix constraints) lie along the same evolutionary path in the complexity parameter space and can be accessed by a variation in their ensemble parameters.

To achieve our objective to derive a *Y*-based formulation of the single-particle entanglement statistics of a typical Hamiltonian represented by Equation (11), we now require a similar formulation for the eigenfunction components. This is briefly reviewed below (the detailed derivation is discussed in [15] and also in the Supplementary Material).

### 4.2. Diffusion Equation for the Joint Probability Density of the Eigenfunctions and Eigenvalues

As the single-particle entanglement measures for a Hamiltonian are defined in terms of the statistics of its eigenfunction components, it is necessary to first derive the *Y*-governed evolution of the latter. This was derived in [15] from an exact route, i.e., an exact diagonalization of the evolution equation for ρ(H). Here we present an alternative approach (although based on second-order perturbation theory, it can be generalized to non-Gaussian cases of ρ(H) too).

Consider an N×N real-symmetric matrix *H* with *O* as the N×N eigenvector matrix of *H* (an orthogonal matrix satisfying $O^{T}O=1$) and *E* as the N×N diagonal matrix of its eigenvalues, $E_{mn}=e_{n}\delta_{mn}$. A small perturbation δH of *H* in the matrix space changes its eigenvalues and eigenfunctions. But as discussed above, with *H* represented by Equation (11), this also results in a complexity parameter-governed evolution of ρ(H) and thereby the joint probability density $P_{ef,ev}\left(\left\{O_{n}\right\},\left\{e_{n}\right\}\right)$ of the eigenvalues and eigenfunctions; here $\left\{O_{n}\right\}$ and $\left\{e_{n}\right\}$ refer to the sets of all eigenfunctions $O_{1},\ldots ,O_{N}$ (the columns of the *U*-matrix) and eigenvalues $e_{1},e_{2},\ldots ,e_{N}$ (the diagonal of the *E*-matrix). The derivation of the *Y*-governed evolution of $P_{ef,ev}$ basically depends on three main steps (a detailed discussion is given in Section SII of the Supplementary Material).

(i) We first determine the first- and second-order moments of $H_{kl}$ from Equation (12). A comparison of the latter with the standard Fokker–Planck equation gives $\langle \delta H_{\mu \nu }\rangle =-\gamma H_{\mu \nu }\delta Y$, $\langle {\left(\delta H_{\mu \nu }\right)}^{2}\rangle =g_{\mu \nu }\delta Y$ and $\langle \left(\delta H_{\mu \nu }\delta H_{\mu^{\prime }\nu^{\prime }}\right)\rangle =0$, with $g_{\mu \mu }=2$ and $g_{\mu \nu }=1$ for μ≠ν. All other averages are of a higher order in δY and can be ignored for a small change in *Y*.

(ii) The information from step (i) is then used, along with second-order perturbation theory for Hermitian operators, to derive the moments of the eigenvalues and eigenfunctions of *H*. We have $\langle \delta O_{jn}\delta O_{kl}\rangle =2v^{2}\left(\sum_{m=1,m\neq n}^{N}\frac{O_{jm}O_{km}\delta_{nl}}{{\left(e_{n}-e_{m}\right)}^{2}}-\frac{O_{jl}O_{kn}\left(1-\delta_{nl}\right)}{{\left(e_{n}-e_{l}\right)}^{2}}\right)\delta Y$ and $\langle \delta O_{jn}\rangle =-v^{2}\sum_{m=1,m\neq n}^{N}\frac{O_{jn}\delta Y}{{\left(e_{n}-e_{m}\right)}^{2}}$, with angular brackets implying conditional ensemble averages with fixed $e_{n},O_{n}$. Similarly, the moments for the eigenvalues can be given as, up to the first order of δY, $\langle \delta e_{n}\delta e_{m}\rangle =8v^{2}e_{n}\delta_{nm}\delta Y$ and $\langle \delta e_{n}\rangle =2\beta v^{2}\left[N_{A}-\frac{\gamma }{\beta v^{2}}e_{n}+\sum_{m=1,m\neq n}^{N}\frac{1}{e_{n}-e_{m}}\right]\delta Y$. Further, to the first order in δY, the ensemble-averaged correlation between $\delta \lambda_{k}$ and $\delta O_{jn}$ is zero (for both β=1 and 2): $\langle \delta e_{k}\delta O_{jn}\rangle =0$.

(iii) Based on a similar dynamics for the matrix elements as indicated by Equation (12), we assume a Markovian dynamics for the eigenvalues and eigenfunctions of *H*. The *Y*-governed evolution of the joint probability density $P_{ef,ev}\left(\left\{O_{n}\right\},\left\{e_{n}\right\}\right)$ can then be described by a standard Fokker–Planck equation,

(17) $$\begin{array}{ccc} \frac{\partial P_{ef,ev}}{\partial Y} & = & \left(L_{U}+L_{E}\right)P_{ef,ev} \end{array}$$

where $L_{U}$ and $L_{E}$ refer to two parts of the Fokker–Planck operator, corresponding to eigenvalues and eigenfunction components, respectively. Here $L_{U}$ is given as

(18) $$\begin{array}{ccc} L_{U}\;\delta Y & = & \sum_{j,n=1}^{N}\frac{\partial }{\partial O_{jn}}\left[\frac{1}{2}\sum_{k,l=1}^{N}\frac{\partial }{\partial O_{kl}}\langle \delta O_{jn}\delta O_{kl}\rangle -\langle \delta O_{jn}\rangle \right] \end{array}$$

and $L_{E}$ is

(19) $$\begin{array}{ccc} L_{E}\;\delta Y & = & \sum_{n}\frac{\partial }{\partial e_{n}}\left[\frac{1}{2}\frac{\partial }{\partial e_{n}}\langle {\left(\delta e_{n}\right)}^{2}\rangle -\langle \delta e_{n}\rangle \right] \end{array}$$

Note here that $P_{ef,ev}$ is subjected to the following boundary condition for ν=1: $P_{ef,ev}\to 0$ for $O_{jn}\to \pm \infty ,\lambda_{n}\to \left[0,\infty \right)$ for j,n=1→N; this follows because the higher-order moments of the ensemble density are assumed to be negligible.

A substitution of the moments in Equation (17) followed by the latter’s integration over all undesired variables will then lead to an evolution equation for the joint probability density of the desired combination of eigenfunction components. This is briefly described below (discussed in detail in [15] and also in Section SIII of the Supplementary Material).

### 4.3. Diffusion Equation for the Joint Probability Density of the Components of a Typical Eigenfunction

The JPDF of the components $O_{nk}$ of an eigenstate, say $O_{k}$ of *H* lying between $\psi_{n}$ and $\psi_{n}+d\psi_{n}$ with n=1→N, can be defined as

(20) $$\begin{array}{c} P_{1}\left(\psi_{1},\ldots ,\psi_{N};Y\right)=\int \delta \left(\Psi -O_{k}\right)P_{ef,ev}D\Omega \end{array}$$

where $D\Omega \equiv \prod_{m<n}\left|e_{m}-e_{n}\right|\prod_{j=1}^{N}De_{j}\prod_{j=1}^{N}DO_{j}$ is the volume element in the eigenvalue–eigenvector space.

A differentiation of Equation (20) with respect to *Y* gives $\frac{\partial P_{1}}{\partial Y}=\int \delta_{\psi ,k}\frac{\partial P_{ef,ev}}{\partial Y}D\Omega$. Using Equation (17) in the integral and repeated partial integrations over the eigenvalues and eigenfunctions other than $\Psi_{n}$ then leads to (see Section SIII of the Supplementary Material)

(21) $$\frac{\partial P_{\psi }}{\partial \Lambda }=\sum_{m,n=1}^{N}\frac{\partial^{2}}{\partial \psi_{n}\partial \psi_{m}}h_{2}+\sum_{n=1}^{N}\frac{\partial }{\partial \psi_{n}}h_{1},$$

with $h_{1}\equiv \left(N-1\right)\psi_{n}P_{\psi }$ and $h_{2}\equiv \left(\delta_{mn}-\psi_{n}\psi_{m}\right)P_{\psi }$. As is clear from the above, the evolution is now governed by

(22) $$\begin{array}{c} \Lambda =\frac{\left(Y-Y_{0}\right)}{\Omega_{e}^{2}} \end{array}$$

with $\Omega_{e}$ as an important system-specific spectral range defined as follows: the eigenvalues at distances more than $\Omega_{e}$ around *e* are uncorrelated. In general, it is of the order of a few local mean level spacings $\Delta_{local}\left(e\right)$ at the energy *e* of interest: $\Omega_{e}\left(e\right)=N_{k}\Delta_{local}\left(e\right)$. Here $N_{k}$ is the number of eigenvalues in the range, intuitively expected to be related to the inverse participation ratio $I_{2}=\frac{1}{N}\sum_{n=1}^{N}{\left|\psi_{n}\right|}^{4}$ at energy *e*, i.e., $N_{k}\propto N\langle I_{2}\rangle$. This suggests that

(23) $$\begin{array}{c} \Omega_{e}\approx \kappa N\langle I_{2}\left(e\right)\rangle \Delta_{local}\left(e\right) \end{array}$$

with κ as the proportionality constant with units of $\langle I_{2}\left(e\right)\rangle$ and $\Delta_{local}\left(e\right)$ as the *local* mean level spacing at energy $e=e_{k}$. In the context of disordered systems, it is believed that $\Omega_{e}$∼$E_{c}$, with $E_{c}$ as the Thouless energy, and is of the order of $\Delta_{local}\left(e\right)$ at energy *e*. We also recall that for many cases, e.g., a *d*-dimensional Anderson Hamiltonian with nearest neighbor hopping, $\Delta_{local}\left(e\right)$ is a ratio of system size *N* and localization volume $\xi^{d}$, with ξ(e) as the average localization length at the energy *e*.

The solution of Equation (21) gives the JPDF of the eigenfunction components for a fixed Λ,N and for an arbitrary initial condition. For finite system sizes, changing system parameters can change Λ continuously between 0 and ∞; this implies, for a finite *N*, the existence of an infinite range of universality classes of the JPDF characterized by continuous values of Λ between 0 and ∞. But as Λ itself is *N*-dependent, a variation in system conditions in an infinite size limit leads to, in general, Λ=0 or ∞, with the solution of Equation (21) approaching the two end points, i.e., the initial state and Wigner–Dyson ensembles, respectively [15]. For specific system conditions, however, a subtle competition between $Y-Y_{0}$ and $\Omega_{e}$ may render Λ independent of *N*; referring to the corresponding Λ value as $\Lambda^{*}$, it would then remain finite even in the N→∞ limit: $\Lambda^{*}=\lim_{N\to \infty }\Lambda \neq 0,\infty$ [15]. The latter in turn implies, in an infinite size limit, the existence of a new intermediate statistics at $\Lambda^{*}$, different from the two end points (i.e., Λ=0 and ∞) and can therefore be referred to as critical. Indeed, based on the complexity of the system, more than one set of system parameters may exist, resulting in different $\Lambda^{*}$ and thereby different critical statistics intermediate between the initial state and Wigner–Dyson ensembles. In contrast to finite *N*, Equation (21) therefore indicates, in the N→∞ limit, the existence of discrete universality classes for the eigenfunction statistics, each characterized by a distinct $\Lambda^{*}$. The explicit appearance of size *N* in Equation (21) (in addition to its implicit appearance through Λ), however, suggests that the statistics at $\Lambda^{*}$ for finite *N* is different from that of infinite *N*.

A detailed study in [13,16] of the eigenvalue dynamics of the ensemble (11) with changing ensemble parameters revealed the existence of a single-parameter formulation of the spectral statistics. Referred as the *spectral complexity parameter*, it is defined as $\Lambda_{e}\left(e\right)=\frac{\left(Y-Y_{0}\right)}{\Delta_{local}^{2}}$ [13,15]. Due to the *N*-dependence of both $Y-Y_{0}$ and $\Delta_{local}$, the spectral statistics shows a finite size scaling as well as a critical behavior at energy *e* if the system conditions lead to an *N*-independent, non-zero, finite $\Lambda_{e}$, referred to as $\Lambda_{e}^{*}$, that is, if the size dependence of $\sqrt{Y-Y_{0}}$ is canceled by $\Delta_{local}$ [16]. Equivalently,

(24) $$\begin{array}{c} \Lambda_{e}^{*}\equiv \lim_{N\to \infty }\Lambda_{e}\left(e\right)=\mathrm{finite}. \end{array}$$

As the correlations among eigenvalues and eigenfunctions are known to be significant in the critical regime, it is useful to express Λ in terms of $\Lambda_{e}\left(e\right)$,

(25) $$\begin{array}{c} \Lambda \left(Y-Y_{0},e\right)=\chi \Lambda_{e}\left(e\right), \end{array}$$

Here χ is again a function of $Y-Y_{0}$ as well as energy *e* (see Section SIII of the Supplementary Material),

(26) $$\begin{array}{ccc} \chi (Y-Y_{0},e) & \approx & \frac{\Delta_{local}^{2}}{\Omega_{e}^{2}}=\frac{1}{\kappa^{2}N^{2}{\langle I_{2}\rangle }^{2}} \end{array}$$

To distinguish it from $\Lambda_{e}$ and $Y-Y_{0}$, Λ hereafter is referred to as the *strength complexity parameter*.

The relation in Equation (25) gives an important insight: it suggests the existence of a finite size scaling of the wavefunction statistics too. The latter in turn manifests through a multifractal behavior of the eigenfunctions at the critical point, with scaling exponents (referred to as critical exponents or multifractal dimensions) dependent on the system parameters [15,22]. As $\Lambda_{e}$ is sensitive to system specifics, the critical (multifractal) exponents can vary from system to system. The occurrence of a critical point and corresponding multifractal behavior of the eigenstates is, however, not a necessary feature of all infinite-size complex systems; based on the Λ-formulation, it requires a specific set of system conditions conspiring with each other and leading to a size-independent Λ (a more detailed discussion of Λ is included in the Supplementary Material).

While the derivation of Equation (21) presented above is based on the second-order perturbation approach for Hermitian matrices and the Markovian assumption, it can be derived exactly by a direct integration of Equation (12) over all eigenvalues and eigenfunctions other than $\Psi_{n}$ [15]. The alternative approach presented here, however, lends further credence to our complexity parameter formulation.

## 5. Evolution of the Single-Particle Entanglement with System Parameters

Writing components $\psi_{n}$ of ψ as $\psi_{n}$ (for notational simplification as well as to retain same notations as used in [15]), we now have $P_{A}=\sum_{n\in A}\psi_{n}^{2}$ and $P_{B}=\sum_{n\in B}\psi_{n}^{2}$. Equation (21) can now be used to derive the first two moments of the SPEE as follows.

### 5.1. Dynamics of SPEE Average

With $S_{A}$ defined in Equation (4), its ensemble average can now be obtained by averaging over the ensemble density $P_{\psi }$ of the eigenfunction ψ,

(27) $$\langle S_{A}\rangle =\int S_{A}P_{\psi }D\psi ,$$

where $D\psi =\prod_{i=1}^{N}d\psi_{i}$. The above definition, along with Equation (21) implying the $\Lambda_{e}$-dependence of $P_{\psi }$, also implies a similar dependence for $\langle S_{A}\rangle$, and its evolution can be derived from Equation (21). The steps are as follows. From Equation (21), we have

(28) $$\frac{1}{\chi }\frac{\partial \langle S_{A}\rangle }{\partial \Lambda_{e}}=\int S_{A}\left(\frac{1}{\chi }\frac{\partial P_{\psi }}{\partial \Lambda_{e}}\right)D\psi ,$$

A substitution of Equation (21) on the right side of the above equation leads to $\frac{1}{\chi }\frac{\partial \langle S_{A}\rangle }{\partial \Lambda_{e}}=I_{1}+I_{2}$, where

(29) $$\begin{array}{ccc} I_{1} & = & \sum_{n}\int S_{A}\left(\frac{\partial }{\partial \psi_{n}}h_{1}\right)P_{\psi }D\psi , \end{array}$$

(30) $$\begin{array}{ccc} I_{2} & = & \sum_{m,n}\int S_{A}\left(\frac{\partial^{2}}{\partial \psi_{n}\partial \psi_{m}}h_{2}\right)P_{\psi }D\psi . \end{array}$$

The above integrals can be solved by repeated partial integrations, but this requires terms dependent on how $S_{A}$ responds to a small change in the eigenfunction components. Here we give the required rates of change for the analysis,

(31) $$\frac{\partial S_{A}}{\partial \psi_{n}}=\left\{\begin{array}{c} -2\psi_{n}\log P_{A}-2\psi_{n},\;n\in A\\ -2\psi_{n}\log P_{B}-2\psi_{n},\;n\in B \end{array}\right.$$

and

(32) $$\frac{\partial^{2}S_{A}}{\partial \psi_{m}\partial \psi_{n}}=\left\{\begin{array}{c} -2\left(1+\log P_{A}\right)\delta_{mn}-\frac{4\psi_{n}\psi_{m}}{P_{A}},\;m,n\in A\\ -2\left(1+\log P_{B}\right)\delta_{mn}-\frac{4\psi_{n}\psi_{m}}{P_{B}},\;m,n\in B \end{array}\right.$$

With repeated partial integration of $I_{1}$ and $I_{2}$ and subsequent use of the relations in Equations (31) and (32), Equation (28) can be further simplified (discussed in detail in Section SV of the Supplementary Material). As our focus here is on the large *N* limit, it can be rewritten as

(33) $$\frac{1}{2\chi N}\frac{\partial \langle S_{A}\rangle }{\partial \Lambda_{e}}+\langle S_{A}\rangle \approx -c_{A}\langle \log P_{A}\rangle -c_{B}\langle \log P_{B}\rangle .$$

with $c_{A}=\frac{N_{A}}{N}$ and $c_{B}=\frac{N_{B}}{N}$. A general solution of the above equation can now be given as

(34) $$\langle S_{A}\rangle =c_{1}e^{-2N\chi \Lambda_{e}}-e^{-2N\chi \Lambda_{e}}\int_{0}^{2N\chi \Lambda_{e}}\left(c_{A}\langle \log P_{A}\rangle +c_{B}\langle \log P_{B}\rangle \right)e^{x}dx.$$

with $c_{1}$ as the constant of integration to be determined from the initial condition. Noting that either $P_{A}=1,P_{B}=0$ or $P_{A}=0,P_{B}=1$ in the separability limit, the terms $\log(P_{A})$ and $\log(P_{B})$ in the integral in Equation (34) diverge at the lower integration limit Λ=0 if the initial state is separable. Choosing a weak separability limit, with $P_{A}=1-\varepsilon ,P_{B}=\varepsilon$ or $P_{A}=\varepsilon ,P_{B}=1-\varepsilon$ with 0<ε≪1, as the initial condition, however, gives $S_{A}\approx \varepsilon \left(1-\varepsilon -\log\varepsilon \right)$ for Λ=0. Equation (34) then implies $c_{1}\approx \varepsilon \left(1-\varepsilon -\log\varepsilon \right)$. Using $\lim_{\varepsilon \to 0}\varepsilon \log\varepsilon \to 0$, this gives $c_{1}=0$ in the limit ε→0.

As is clear from Equation (33), the determination of the Λ-dependence of $\langle S_{A}\rangle$ requires prior knowledge of the similar dependence of $\langle \log P_{A}\rangle$ and $\langle \log P_{B}\rangle$. But this in turn requires knowledge of the negative moments of $P_{A}$ and leads to a set of hierarchical equations with no available solution (this can be seen by following the similar steps used for the derivation of $S_{A}$). To overcome this technical difficulty, the only option available at this stage is to consider a crude approximation, i.e., replace $\langle \log P_{A}\rangle \approx \log\langle P_{A}\rangle$ and similarly for $P_{B}$ in Equation (33) (the approximation is often used for partition function studies of systems with annealed randomness). The Λ-dependence of $\langle P_{A}\rangle$ can now be determined, proceeding as in the $\langle S_{A}\rangle$-case. By definition, we have

(35) $$\begin{array}{c} \langle P_{A}\rangle =\int P_{A}P_{\psi }D\psi , \end{array}$$

(36) $$\begin{array}{c} \Rightarrow \frac{1}{\chi }\frac{\partial \langle P_{A}\rangle }{\partial \Lambda_{e}}=\int P_{A}\left(\frac{1}{\chi }\frac{\partial P_{\psi }}{\partial \Lambda_{e}}\right)D\psi , \end{array}$$

where $D\psi =\prod_{i=1}^{N}d\psi_{i}$. Substitution of Equation (21) in the above equation gives

(37) $$\frac{1}{\chi }\frac{\partial \langle P_{A}\rangle }{\partial \Lambda_{e}}=\sum_{m,n}\int P_{A}\left(\frac{\partial^{2}h_{2}}{\partial \psi_{n}\partial \psi_{m}}\right)P_{\psi }D_{z}+\sum_{n}\int P_{A}\left(\frac{\partial h_{1}}{\partial \psi_{n}}\right)P_{\psi }D_{z}.$$

Further, by repeated partial integration and realizing that the non-zero terms after differentiation in $\sum_{m,n}$ are such that m,n∈A, we get

(38) $$\frac{1}{\chi }\frac{\partial \langle P_{A}\rangle }{\partial \Lambda_{e}}=2\left(N_{A}-N\langle P_{A}\rangle \right).$$

As a check, we note that, for the balanced bipartition case $N=2N_{A}$, the above equation gives expected limiting behavior $\langle P_{A}\rangle \to 1/2$ as Λ→∞. Assuming the separability limit as the initial condition, the solution of Equation (38) can be given as

(39) $$\langle P_{A}\left(\Lambda_{e}\right)\rangle =\frac{1}{2}\left[1+e^{-4\chi N_{A}\Lambda_{e}}\right].$$

With $P_{B}=1-P_{A}$, the above equation gives $\langle P_{B}\left(\Lambda_{e}\right)\rangle =\frac{1}{2}\left[1-e^{-4N_{A}\chi \Lambda_{e}}\right]$ and $\langle P_{A}\rangle$∼1 and 1/2 for $\chi \Lambda_{e}<1/\left(4N_{A}\right)$ and $\chi \Lambda_{e}>1/\left(4N_{A}\right)$, respectively, thus indicating a rapid transition as a function of $\Lambda_{e}$ with $\chi \Lambda_{e}=1/\left(4N_{A}\right)$ as the point of inflection. A smooth crossover around this point can then be seen as a function of $N_{A}\chi \Lambda_{e}$; here we recall that χ, defined in Equation (26), also changes with $Y-Y_{0}$.

Substitution of $\langle \log\left(P_{A}\right)\rangle \approx -\log2+e^{-4\chi N_{A}\Lambda_{e}}$ and $\langle \log\left(P_{B}\right)\rangle \approx -\log2-e^{-4\chi N_{A}\Lambda_{e}}$ in Equation (33) now gives

(40) $$\begin{array}{ccc} \langle S_{A}\rangle & = & \log2(1-e^{-4\chi N_{A}\Lambda_{e}}), \end{array}$$

with χ defined in Equation (26). As χ depends on the system parameters, taking different values in localization and delocalization regimes, this in turn leads to two possible solutions. But as is clear from the above, a rescaling of $\Lambda_{e}$ by χ is expected to map one solution on the other.

**Figure 2:**
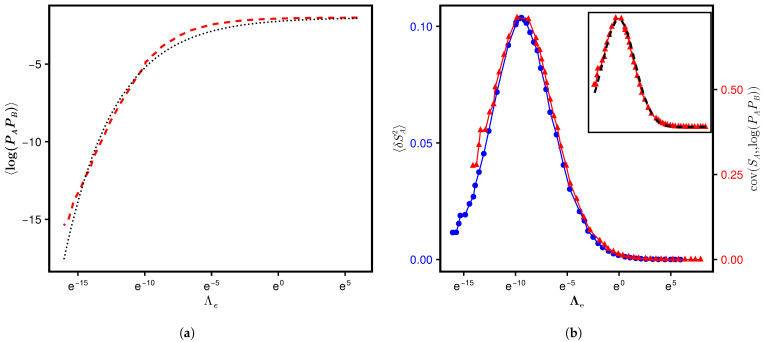
Λ-dependence of $\log(P_{A}P_{B})$: The figure displays the $\Lambda_{e}$-governed evolution of (**a**) $\langle \log\left(P_{A}P_{B}\right)\rangle$ and (**b**) a comparison of the variance $\langle \delta S_{A}^{2}\rangle$ (blue dots) and the covariance $cov(S_{A},\log\left(P_{A}P_{B}\right))$ (red triangles) (in base $\log_{2}$) for a cubic Anderson lattice of linear size L=12 for different combinations of system parameters. As the statistical uncertainties here are small compared to symbol size, we do not display the error bars to improve clarity. The numerical results confirm our theoretical conjectures, namely, Equations (41) (black dotted line in (**a**)) and (46). The inset in panel (**b**) displays the good agreement of Equation (48) (black dashed line) with numerics. The solid lines connecting the symbols are guides to the eye only and do not represent any fitting.

As discussed in the next section, while the numerical result for $\langle S_{A}\rangle$ has the same form as the one given in Equation (40), it does not exactly match. We believe this discrepancy arises from the approximation 〈logx〉≈log〈x〉 (based on the numerics for $x\equiv P_{A}$ discussed in the next section, the approximation seems to be good for large Λ). To rule out the possibility that the discrepancy arises from the approximation 〈logx〉≈log〈x〉, we consider the following intuitive approach to improve our theoretical prediction. For small Λ ranges, assuming that $P_{A},P_{B}$ are perturbed slightly from their Λ=0 values, i.e., $P_{A}=1-\varepsilon ,P_{B}=\varepsilon$ with ε≪1, we have $\log(P_{A}P_{B})\approx \log\varepsilon -\varepsilon$. For large Λ ranges where typically $P_{A}=\left(1/2\right)-\varepsilon ,P_{B}=\left(1/2\right)+\varepsilon$, we have $\log(P_{A}P_{B})\approx -2\log2$. This suggests a rapid decay of $\log(P_{A}P_{B})$ from a divergence to a constant value with increasing Λ. Based on the above insights, we conjecture, for the balanced case ($N_{A}=N_{B}$) and for Λ-governed evolution of $\langle \log\left(P_{A}P_{B}\right)\rangle$ from a separable initial state, that

(41) $$\begin{array}{c} \langle \log\left(P_{A}P_{B}\right)\rangle =-\left(2\log2+b_{0}\Lambda^{\nu -1}\right) \end{array}$$

with ν>0. The above conjecture is in agreement with the balanced case numerics for ν=0.74 and $b_{0}=0.24$ (Figure 2, numerical details discussed in the next section). As a measure of goodness of fit, we find that the $R^{2}$ value (defined in Appendix B) for the above conjecture in Figure 2 is about 99% and the standard error in determining parameters ν and $b_{0}$ is about 0.007 and 0.024, respectively. Substitution of Equation (41) in Equation (34) gives (for the $N_{A}=N_{B}$ case)

(42) $$\begin{array}{ccc} \langle S_{A}\rangle & = & \frac{1}{2}e^{-4\chi N_{A}\Lambda_{e}}\int_{0}^{4\chi N_{A}\Lambda_{e}}\left(2\log2+b_{0}x^{\nu -1}\right)e^{x}dx. \end{array}$$

(43) =log2(1−e−4χNAΛe)+b02B(1,ν)(4χNAΛe)νF11ν,ν+1,4χNAΛee−4χNAΛe

with B(a,b) as the Beta function and F11(a,b;x) as the confluent Hypergeometric function. As discussed in the next section, notwithstanding a good agreement between $\langle \log\left(P_{A}P_{B}\right)\rangle$ numerics and the conjecture in Equation (41), a comparison of Equation (43) with $\langle S_{A}\rangle$-numerics does not show any significant improvement over Equation (40).

For Λ→∞, the evolution approaches the steady state with $\frac{\partial \langle S_{A}\rangle }{\partial \Lambda_{e}}=0$ with $\langle P_{A}\rangle =\langle P_{B}\rangle =1/2$. Equation (40) then gives ${\langle S_{A}\rangle }_{\infty }=\log2$. This is consistent with the prediction based on Equation (4). This also lends credence, indirectly, to the approximation $\langle \log P_{A}\rangle \approx \log\langle P_{A}\rangle$ and similarly for $P_{B}$ used in Equation (33).

### 5.2. Dynamics of SPEE Variance

The evolution equation for the variance can similarly be obtained. Here we give the equation for the simple case $N_{A}=N/2$ (also used in our numerics),

(44) $$\frac{1}{4\chi N}\frac{\partial \langle {\left(\delta S_{A}\right)}^{2}\rangle }{\partial \Lambda_{e}}=-\langle {\left(\delta S_{A}\right)}^{2}\rangle -\mathrm{cov}\left(S_{A},\log\left(P_{A}P_{B}\right)\right),$$

where $\mathrm{cov}\left(S_{A},\log\left(P_{A}P_{B}\right)\right)\equiv \langle S_{A}\log\left(P_{A}P_{B}\right)\rangle -\langle S_{A}\rangle \langle \log\left(P_{A}P_{B}\right)\rangle$. The variance of the SPEE is thus determined by the covariance of $S_{A}$ and $\log(P_{A})$. Proceeding again as in the case of Equation (33), we obtain

(45) $$\langle {\left(\delta S_{A}\right)}^{2}\rangle =c_{2}e^{-4\chi N\Lambda_{e}}-e^{-4\chi N\Lambda_{e}}\int_{0}^{4\chi N\Lambda_{e}}\left(\mathrm{cov}(S_{A},\log\left(P_{A}P_{B}\right))\right)e^{x}dx.$$

with $c_{2}$ as the constant of integration to be determined from the initial condition. A choice of the separability condition as the initial condition implies $\langle {\left(\delta S_{A}\right)}^{2}\rangle =0$ for the initial state; Equation (45) then implies $c_{2}=0$.

As in the case of $\langle S_{A}\rangle$, here again determination of $cov(S_{A},\log\left(P_{A}P_{B}\right))$ involves negative moments and requires solving a hierarchical set of equations, which is technically complicated. This motivates us again to consider an intuitive approach. As mentioned above, for $P_{A}=1-\varepsilon ,P_{B}=\varepsilon$ with ε≪1 (which is the case for small Λ ranges), we have $S_{A}\approx \varepsilon \left(1-\varepsilon -\log\varepsilon \right)\approx \varepsilon$ and $\log(P_{A}P_{B})\approx \log\varepsilon -\varepsilon$. This gives $S_{A}^{2}$∼$-S_{A}\log\left(P_{A}P_{B}\right)$. This is also valid for the case with $P_{B}=1-\varepsilon ,P_{A}=\varepsilon$. For large Λ ranges where typically $P_{A}=\left(1/2\right)-\varepsilon ,P_{B}=\left(1/2\right)+\varepsilon$, we have $S_{A}\approx \log2+\left(1-2\varepsilon \right)\varepsilon -\left(1+2\varepsilon \right)\varepsilon =\log2-4\varepsilon^{2}$ and $\log(P_{A}P_{B})\approx -2\log2$; this again suggests that $S_{A}^{2}$∼$-S_{A}\log\left(P_{A}P_{B}\right)$. Based on the above insights, we conjecture that $\mathrm{cov}\left(S_{A},\log\left(P_{A}P_{B}\right)\right)\left(\Lambda \right)$ as a function of Λ has the same mathematical form as ${\left(\delta S_{A}\right)}^{2}$ as a function of θΛ (with $\Lambda =\chi \Lambda_{e}$):

(46) $$\begin{array}{c} \mathrm{cov}\left(S_{A},\log\left(P_{A}P_{B}\right)\right)\left(\Lambda \right)\approx {\left(\delta S_{A}\right)}^{2}\left(\theta \Lambda \right) \end{array}$$

with θ as a rescaling factor. As displayed in Figure 2, the conjecture is corroborated by our numerics for the Anderson case (system details given in Section 6), which gives

(47) $$\begin{array}{c} \langle {\left(\delta S_{A}\right)}^{2}\rangle \approx a\exp\left[-b\left(\log{\left(c\Lambda \right)}^{2}\right)\right], \end{array}$$

(48) $$\begin{array}{c} \mathrm{cov}\left(S_{A},\log\left(P_{A}P_{B}\right)\right)\approx a\exp\left[-b\left(\log{\left(\theta c\Lambda \right)}^{2}\right)\right] \end{array}$$

with θ=7. As can be checked by a direct substitution, the above functional forms are consistent with Equation (45) too. Here, $c=10^{4}$ is a rescaling of Λ to match the mean with the numerics, and b=0.06 and 0.05 for the variance and covariance, respectively. The absolute error in the determination of the normalization factor *a* from its exact value $\sqrt{b/\pi }$ is about 0.036 and 0.58 for the variance and the covariance curves, respectively.

### 5.3. Relevance of Λ and Complexity Constants

Equations (34) and (45) describe the common mathematical formulation for the SPEE statistics of a typical eigenfunction of the Hamiltonians modeled by Equation (11), with information about the ensemble parameters and thereby system parameters appearing through $\Lambda =\chi \Lambda_{e}$ only. This implies that the SPEE statistics respond to changing system parameters in a collective way instead of individually to each of them. The information about crucial system parameters may appear through $Y-Y_{0}$ as well as $R_{local}$. For example, the dimensionality and boundary conditions both influence the basis connectivity, i.e., degree of sparsity of the matrix, which is also reflected in the distribution parameters v,b. The dimensionality also affects $R_{local}$ through the average localization length $\xi^{d}$ (the sensitivity of $\Lambda_{e}$ to the system parameters of Anderson Hamiltonians is discussed in detail in [15,16]).

The system size *N* is another important parameter that affects the evolution of the eigenfunction statistics significantly and thereby the related measures. The dependence on *N* can, however, vary from one measure to another [15]. Indeed, in contrast to Equation (21), the size *N* appears in SPEE statistics through $\chi N_{A}\Lambda_{e}$. For large $N_{A}$, the statistics is therefore expected to approach the separable state behavior in the limit $\chi N_{A}\Lambda_{e}\ll 1$ and the maximum SPEE in the limit $\chi N_{A}\Lambda_{e}\gg 1$. For $\Lambda_{e}=1/N_{A}\chi$, the SPEE statistics is predicted to become size-independent and remains different from both the separable state as well as the maximum entangled state, even in an infinite size limit.

Equations (34) and (45) describe the evolution of the SPEE statistics from a separable initial state at Λ=0. While the latter can arise from different sets of initial conditions for different systems, their path of the evolution is fixed by the complexity constants. Thus, in general, SPEE statistics for different Hamiltonians may evolve along different paths in the complexity parameter (t)-space. It is, however, possible that two different Hamiltonians, represented in two different bases, still have the same sets of complexity constants $t_{2},\ldots ,t_{M}$ for both of them. Typical eigenstates of such Hamiltonians will evolve, with Λ as the evolution parameter, along the same path in *t*-space, but again that does not ensure same SPEE statistics for them. As discussed in the previous section, the latter requires an analogy of Λ and can further be explained as follows: Consider two different systems labeled as “1” and “2”, both represented by the ensemble (11), with their different system conditions giving rise to different sets of ensemble parameters labeled as “$x_{1}$” and “$x_{2}$”. If the physical conditions in “1” and “2” are such that the relation

(49) $$\begin{array}{c} \Lambda_{1}\left(x_{1},e_{1}\right)=\Lambda_{2}\left(x_{2},e_{2}\right)\equiv \Lambda \end{array}$$

is fulfilled, the statistical behavior of system “1” at energy $e_{1}$ is predicted to be analogous to that of “2” at energy $e_{2}$. The above analogy, however, need not imply an analogy for previous histories of the two systems (indeed they may show completely different behavior for $0<\Lambda_{x1},\Lambda_{x2}<\Lambda_{x}$) or for their future or even at other energies. We emphasize that the above universality is applicable for the Hamiltonian matrices belonging to same matrix constraint class, e.g., real-symmetric or complex Hermitian, etc., which in turn depends on the underlying symmetries and conservation laws (referred to as global constraints) [21]. (We recall that the matrices belonging to same matrix constraint class may belong to ensembles with different ensemble parameters (referred to as ensemble constraints or “local” constraints).

A relevant query in the above context is whether the complexity constants $t_{2},\ldots ,t_{M}$ can always be chosen the same for different Hamiltonian ensembles (of type represented by Equation (11)) if they are subjected to the same set of global constraints. Intuitively the answer is in the affirmative and can be justified as follows: a physically motivated basis to represent a Hamiltonian is usually based on its global constraints, e.g., symmetry conditions and conservation laws, which the chosen basis is expected to preserve under perturbation of the system. For example, for a time-reversal Hamiltonian, an appropriate basis is the one in which the Hamiltonian matrix is real-symmetric. Different Hamiltonians with a common set of global constraints can then be represented in same/ equivalent basis if their dynamics of interest preserves them. For example, both Anderson and RP Hamiltonians discussed in Section 3 are time-reversal invariant but subjected to no other symmetry constraint. As our interest is in their evolution, which at no stage violates the time-reversal symmetry, both can then be well represented by real-symmetric matrices (this is, however, not the case, e.g., if the Hamiltonian dynamics violates the time-reversal symmetry or is subjected to column constraints). A typical matrix, represented in a fixed basis, in general has many basis constants [23,24], which can then be chosen as the complexity constants $t_{2},\ldots ,t_{M}$. This is further explained through two examples (namely Anderson and RP) in Section SV of the Supplementary Material. (It is worth reemphasizing that two different Hamiltonians, both represented by real-symmetric matrices, need not belong to ensemble ρ(H) (Equation (11)) with analogous (v,b) sets; indeed the relative matrix elements’ strengths and the ensemble parameters depend on the local constraints, e.g., disorder, interaction, etc. This in turn results in different complexity parameters, e.g., $\Lambda_{e}$, for them, even though their complexity constants are same. While a variation in their respective system conditions may force such Hamiltonians to follow the same evolutionary path in complexity space, their statistics in general need not be same.

## 6. Numerical Analysis

The theoretical analysis described in the previous section predicts the existence of an infinite range of universality classes of SPEE statistics, characterized by the complexity parameter Λ, among the eigenstates if their Hamiltonians are represented by Equation (11) and belong to same symmetry class. The robustness of our theoretical prediction makes it imperative to seek their numerical verification. Here we numerically analyze the two ensembles described by Equations (8) and (9).

**Anderson Ensemble (AE):** We exactly diagonalize the N×N Hamiltonian matrix, represented in the site basis, of a three-dimensional cubic Anderson lattice (Equation (5)) subjected to a periodic boundary condition for two linear lattice sizes L=12 and 18 (with $N=L^{3}$). The matrix for each *N* is considered for several on-site disorder strengths *w*, off-diagonal isotropic random hopping with variance $w_{1}$ (with its mean *t* fixed to 1/2), and the hopping range parameter *k*. Here k=1 implies hopping to six nearest neighbours (z=6) and k=2 implies hopping to twenty-four next nearest neighbours (z=24). Throughout this analysis, the hopping rate is kept fixed to 1/2. Equations (8) and (16) give the *ensemble complexity parameter* for the case $Y=-\frac{N}{2M\gamma }A+C_{0}$, with $A=\ln|1-\gamma w^{2}|+\left(z/2\right)\ln[|1-2\gamma w_{1}^{2}||t+\delta_{t,0}{|}^{2}]$, and $M=\frac{N}{2}\left[N+z\left(1-\delta_{t,0}\right)+1\right]$, with γ=1/2. The initial condition of the Hamiltonians in the ensemble is chosen to be the localized phase such that $w=w_{m}\gg 1$. Thus, we have

(50) $$Y-Y_{0}=-\frac{N}{2M\gamma }\left(A-A_{0}\right)$$

with $A_{0}$ as the A value corresponding to the initial state. The *spectral complexity parameter*$\Lambda_{e}$ for this case can now be calculated from Equation (25), with $\Delta_{local}\left(e\right)=\frac{\Delta_{e}N}{\xi^{d}}$ [15] (also discussed in Section SVII of the Supplementary Material). To determine the average localization length $\xi^{d}$, here, we use the relation $\xi^{d}\approx {\langle I_{2}\rangle }^{-1}$ (valid in the spectrum bulk near e=0), with $I_{2}$ as the inverse participation ratio (IPR) of the eigenfunction of interest, and $\Delta_{e}$ as the mean level spacing; both $\langle I_{2}\rangle$ and $\Delta_{e}$ are determined numerically.

For each set of parameters $w,w_{1},k$, we consider an ensemble of $M=10^{3}$ disorder realizations and use the standard *shift-invert* diagonalization technique to calculate 1% of the total eigenfunctions in a small range δE at the energy E=0 [25,26]. This in turn gives the total NM/100 eigenfunctions for an average analysis of SPEE for a given set of system conditions; the averaging here can then consist of both ensemble as well as spectral averaging. An important point worth emphasizing here regards the choice of spectral range δE: based on our theoretical predictions, Λ(e) (Equation (25)) governs the SPEE statistics and its quantitative analogy is necessary to observe the analogy of the statistics for different parametric combinations. But Λ is energy-dependent too, which makes it necessary in principle to consider only ensemble averaging. To improve the statistics, however, it may be imperative to consider a spectral averaging over a small optimized range δE such that Λ does not vary significantly over the range.

**Figure 3:**
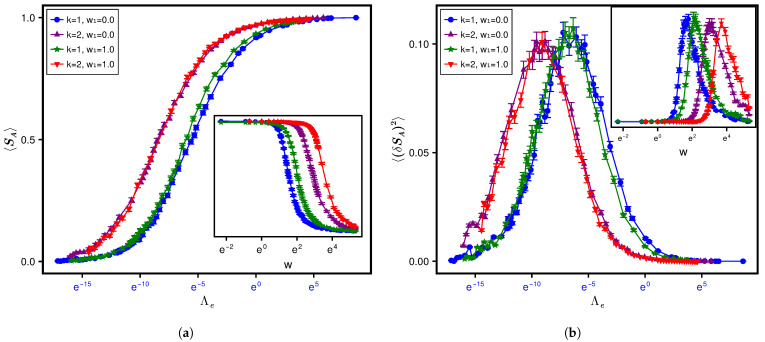
Role of χ in Λ-governed evolution: To illustrate the effect of ignoring the parameter χ in Equation (25) for Λ, the figure displays the $\Lambda_{e}$-governed evolution of (**a**) average $\langle S_{A}\rangle$ and (**b**) variance $\langle \delta S_{A}^{2}\rangle$ (in the $\log_{2}$ base) along with error bars for a cubic Anderson lattice of linear size L=12 for different combinations of other system parameters (for both random and non-random hopping $w_{1}$ and the nearest neighbors (k=1) as well as the next nearest neighbors (k=2) while keeping t=0.5 fixed). The evolution of the SPEE measures for k=2 is now visually shifted from k=1, but as is clear from the Figure 4, the two curves can be made to collapse onto a single curve by a rescaling. For comparison, the inset also displays the evolutions in terms of diagonal disorder *w*.

For calculating the SPEE measures, we consider a horizontal bipartition of the lattice through the center (Figure 1) such that $N_{A}=N_{B}=\frac{N}{2}$. Throughout the numerical analysis, we have used log base 2 to calculate the SPEE and its statistics. Furthermore, the figures, except Figure 1, are shown on a semi-log plot. To see the effect of the type of averaging, we analyze the results both ways, i.e., (i) ensemble averaging only and (ii) ensemble–spectral averaging (over various disorder realizations and over about 1% of the total eigenstates per realization at E=0) for a fixed set of system parameters. The above analysis is repeated by varying the parameters, which gives us the average and variance of SPEE for many Λ values. Our analysis indicates no significant difference in the results obtained by the two types of averaging. This is, however, due to the choice of the eigenfunction for the analysis; we have analyzed the eigenfunction at E=0. (The local spectral density and, as a result, $\Lambda_{e}$ for AE does not vary significantly within a small energy range around E=0 [16].) To avoid repetition of almost the same figures, here we include the figures corresponding to ensemble averaging only. The results for $\langle S_{A}\rangle$ and its variance are displayed in the top and bottom panels in Figure 3.

**Figure 4:**
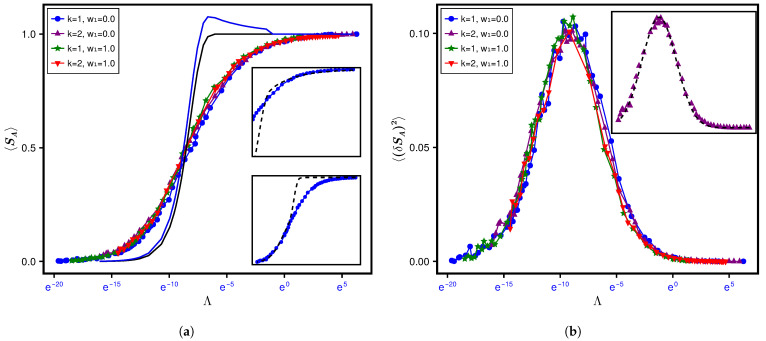
Evolution of SPEE measures for an Anderson lattice (case L=12): The evolution of (**a**) the average $\langle S_{A}\rangle$ and (**b**) the variance $\langle \delta S_{A}^{2}\rangle$ is shown. While the other details here are same as in Figure 3, the evolution parameter is now Λ. The error bars, being very small, are no longer displayed to improve clarity. The convergence of all curves to single curve in the main panel and its lack in the insets of Figure 3 reveal the role of $\Lambda =\chi \Lambda_{e}$ as the primary evolution parameter. The black and blue solid lines in the $\langle S_{A}\rangle$-plot depict our theoretical predictions, Equation (40) and Equation (42), respectively. The insets in panel (a) display a comparison with two fitted functions (black dashed line), (i) $\langle S_{A}\rangle \approx \left(1-e^{-2N\Lambda }\right)+{\left(2N\Lambda \right)}^{1/2}e^{-8N\Lambda }$ and (ii) $\langle S_{A}\rangle \approx \left(1-e^{-2N\Lambda }\right)-0.36{\left(2N\Lambda \right)}^{-0.26}$ ((i) fits well in a small Λ range and (ii) in a large Λ range). The inset in panel (b) shows a comparison of numerics with Equation (47) (black dashed line).

To illustrate the relevance of $\Lambda =\chi \Lambda_{e}$ as a single parameter governing the SPEE dynamics (instead of $\Lambda_{e}$, which plays that role for spectral statistics [16]), it is important to first understand what happens if the dynamics is studied in terms of one of the system parameters. The insets in the top and bottom panels of Figure 3 display the variation with respect to diagonal disorder *w* for a fixed lattice size L=12 (i.e., $N=L^{3}=1728$) but for four different combinations of $w_{1}$ and *k*. As can be seen from both panels of the figure, the curves corresponding to lattices with nearest neighbour interaction (z=6, denoted by k=1) but with different hopping types (random hopping with off-diagonal variance $w_{1}=1$ as well as non-random hopping $w_{1}=0$ both for fixed hopping strength t=1/2) collapse onto a single curve in terms of $\Lambda_{e}$. The curves, however, collapse to a different curve if the next nearest neighbour interaction (z=24, denoted by k=2) is switched on while keeping the hopping variances the same ($w_{1}=1$ and $w_{1}=0$). This is consistent with the Λ-based formulation: the SPEE statistics for two different system parameter sets is expected to be analogous only in terms of Λ and not $\Lambda_{e}$. As the ensemble-averaged localization length ξ and thereby $\Delta_{local}$ for the nearest neighbor differs significantly from that of next neighbour hopping, χ for the two cases is expected to be a different constant, which in turn leads to two different curves. To determine Λ, however, prior knowledge of χ for the k=1 and 2 cases is needed. The numerical analysis for L=12 gives an analogous $\langle I_{2}\rangle$-variation with *w* for the two cases (see Figure S1 in the Supplementary Material). Equation (26) then implies $\frac{\chi_{2}}{\chi_{1}}=\frac{\kappa_{1}^{2}}{\kappa_{2}^{2}}$, with subscripts 1 and 2 implying the variable for cases k=1 and 2, respectively. Due to a lack of exact information about $\kappa_{1}$, $\kappa_{2}$, we conjecture that $\kappa_{2}=\frac{\kappa_{1}}{2\sqrt{d}}$ (based on numerical estimation); the latter suggests that $\chi_{2}\approx 12\chi_{1}$. Indeed, as can be seen from Figure 4, a rescaling of $\Lambda_{e}$ for the k=1 case by a factor of 12 (keeping all other details the same as in Figure 3) leads to convergence of the corresponding statistics to the k=2 case, thereby confirming a single-parametric evolution in terms of Λ. The display in Figure 3 also provides another insight, i.e., an insensitivity of χ to $w_{1}$ (as revealed by the analogy of $\Lambda_{e}$-governed curves for a fixed *k* but two different $w_{1}$ values); this insight may be potentially relevant for a theoretical χ-formulation in the future.

**Figure 5:**
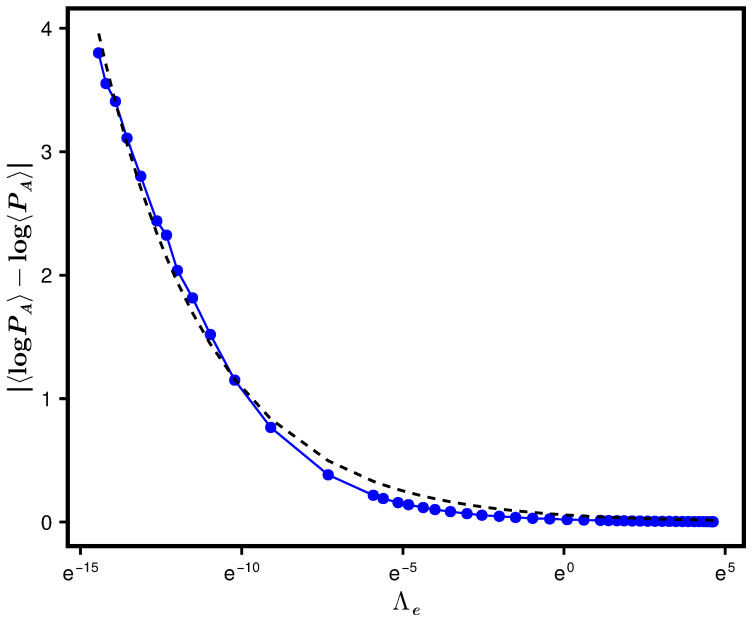
**Λ dependence of the difference $|\langle \log P_{A}\rangle -\log\langle P_{A}\rangle |$:** To understand the limit of the validity of the approximation $\langle \log P_{A}\rangle =\log\langle P_{A}\rangle$, the figure displays their absolute difference for a fixed set of system parameters ($k=2,w_{1}=1.0,L=12$) used in our SPEE analysis. The dashed line displays the theoretical fit $0.059\Lambda_{e}^{-0.29}$. The solid line connecting the symbols is guide to the eye only and do not represent any fitting. Although the latter implies that the approximation is valid only in the large $\Lambda_{e}$ regime, the theoretical result for $\langle S_{A}\rangle$ based on the approximation is qualitatively close to the functional form revealed by numerics (the latter obtained by the exact SPEE formula).

In the previous section, we predicted Equation (40) as a theoretical formulation for $\langle S_{A}\rangle$, assuming $\langle \log P_{A}\rangle \approx \log\langle P_{A}\rangle$. Figure 4 displays a comparison of the numerics based on the exact SPEE formulation (defined in Equation (4)) with Equation (40). While the latter seems to predict an almost similar functional form for $\langle S_{A}\rangle$, a quantitative deviation between the numerics and approximate theory is clearly visible. The latter is expected because $\langle \log P_{A}\rangle \approx \log\langle P_{A}\rangle$ seems to be a good approximation only in a large Λ range (as displayed in Figure 5 for one set of parameters, we find that $|\langle \log P_{A}\rangle -\log\langle P_{A}\rangle |\approx 0.059\Lambda_{e}^{-0.29}$). To improve the prediction, we conjectured Equation (41) for $\langle \log\left(P_{A}P_{B}\right)\rangle$ for the case $N_{A}=N_{B}$, which in turn led to Equation (43) for $\langle S_{A}\rangle$. A comparison with $\langle \log\left(P_{A}P_{B}\right)\rangle$ numerics displayed in Figure 2 shows a good agreement with Equation (41) for ν=0.74 and $b_{0}=0.24$. This motivates us to compare the $\langle S_{A}\rangle$ numerics with Equation (43) using the same ν and $b_{0}$. As displayed in Figure 4, however, Equation (43) does not lead to any significant improvement over Equation (40). We believe that the deviation could be an artifact of computations on the logarithmic scale as well as finite system sizes, while the theoretical predictions are derived in the large *N*-limit. We also considered two fitted functions for large and small Λ; these results are also displayed in the inset of Figure 4.

**Figure 6:**
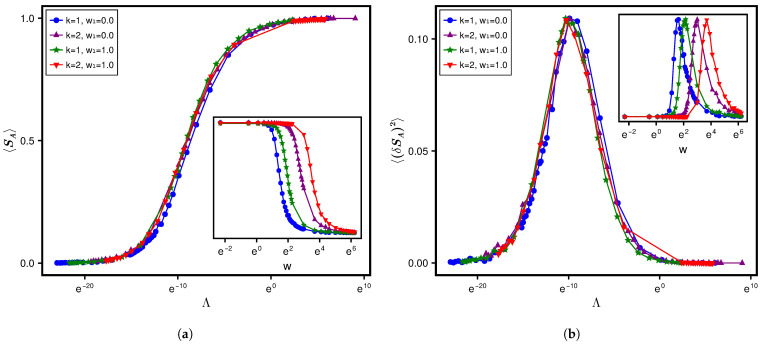
Evolution of SPEE measures for an Anderson lattice (case L=18): The SPEE statistics is now displayed for L=18 (i.e., N=5832), with (**a**) the average $\langle S_{A}\rangle$ and (**b**) the variance $\langle \delta S_{A}^{2}\rangle$, while keeping other parameter details the same as in Figure 3. Here again the statistical uncertainties are small compared to symbol size, making error bars irrelevant. In agreement with Figure 4, the curves again converge to a single curve in the main panel as a function of Λ. A comparison of this figure with Figure 4 also indicates the analogy of the statistics for L=18 and L=12 as a function of Λ. With statistical uncertainties small compared to symbol size here again, we do not display the error bars to retain clarity.

**Figure 7:**
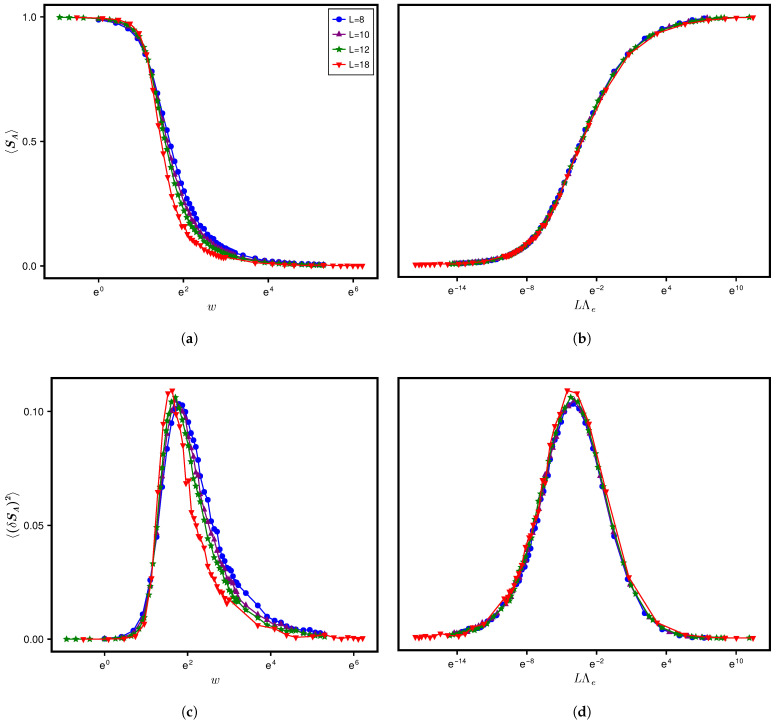
Finite size scaling in SPEE statistics: The panel (**a**) of the figure illustrate the finite size scaling of the average SPEE and (**c**) its variance for a typical state of the N×N Anderson Hamiltonian in terms of diagonal disorder *w* and under the balanced condition $N_{A}=N_{B}$ for k=1 and $w_{1}=0.0$. Here $N=L^{3}$, with *L* as the linear size of the cubic Anderson lattice subject to periodic boundary conditions. While the curves for different system sizes are quite close here, their crossing at $w=w_{c}\approx 3$ is clearly visible. In contrast, the panel (**b**) of the figure illustrates the evolution of the average SPEE and (**d**) its variance, with $L\Lambda_{e}$ as an evolution parameter. The collapse of all curves into a single one as a function of $L\Lambda_{e}$ indicates the latter as the only relevant scale.

In order to understand the effect of system size on SPEE statistics, we consider its dynamics for a larger lattice size L=18 (i.e., $N={\left(18\right)}^{3}$) while keeping the other parameter details the same as in Figure 3. However this time, since we generated only 100 samples, we use both ensemble and spectral averaging. The corresponding Λ-governed SPEE behavior (for N=5832) is displayed in Figure 6. Similarly to Figure 3, the curves again converge to a single curve in the main panel as a function of Λ (while a lack of convergence in the inset for *w*-governed evolution is still visible). A comparison of the display in the main panels of Figure 6 for L=18 and Figure 4 for L=12 also indicates an analogy of their statistics as a function of Λ (also displayed in Figure 7 for a single set of system conditions).

While Figure 4 and Figure 6 validate the Λ formulation of the SPEE statistics for four different $w_{1},k$ combinations, the computational limitations permit us to analyze each combination only for two fixed system sizes. It is therefore natural to query the finite size effects on the Λ formulation and whether it remains valid even in an infinite size limit. We recall that theoretically a finite Λ value in the limit N→∞ implies a critical wavefunction statistics different from the initial state and the ergodic limit (discussed in Section 4.3). We also recall, with $\Delta_{local}\left(e\right)$ given below Equation (50), that $\Lambda_{e}$ for a *d*-dimensional Anderson lattice of linear size *L* depends on the ratio $\frac{N}{\xi^{d}}$ (with $N=L^{d}$), thereby implying a finite size scaling of the spectral statistics (verified in [16]). This in turn suggests, from Equation (25), a finite size scaling of the wavefunction statistics too, although the relevant length scale, say $\xi_{\mathrm{ent}}$, competing with system size *L* need not be same as the average localization length ξ of the eigenfunctions at energy *e*. Intuitively this can be explained as follows. With usual eigenfunction correlation measures based on the intensities at a few sites, their scaling as a function of $\frac{L}{\xi }$ can be explained as a competition between the tendency to spread over whole basis space and the tendency to localize to a few basis states. In the SPEE case, however, the collective response of the intensities in each subsystem affects the correlations between them. Noting that the intensities in a subsytem need not belong to neighboring basis states, the emergence of new length scales dominating such quantum correlations is therefore not unexpected. While ξ is the relevant length scale for the localization-to-delocalization transition in the basis space, $\xi_{ent}$ is expected to play a similar role for the separability-to-maximum entanglement transition between the two subsystems. Indeed, as displayed in Figure 7, a comparison of SPEE statistics as a function of *w* (for a fixed $w_{1},k$ combination), for four different system sizes $N=L^{3}$ with L=8,10,12,18 and under the balanced condition $N_{A}=N_{B}$, indicates a finite size scaling, with a critical disorder $w_{c}\approx 3$. Although the curves for different system sizes are quite close here (seemingly an artifact of the balanced condition $N_{A}=N_{B}$ chosen in our analysis), their crossing at $w=w_{c}$ is clearly visible; the latter therefore marks the critical point of the separability-to-maximum entanglement transition. The determination of $\xi_{ent}$ and its relation with ξ requires, however, a detailed theoretical as well as numerical analysis and is currently under study.

Figure 7 reveals another interesting insight: $\langle S_{A}\rangle$ behavior for all *N* values collapse onto a single curve as a function of $L\Lambda_{e}$, thereby indicating that the statistics are governed only by $L\Lambda_{e}$ (similarly for the $S_{A}$-variance) irrespective of system size. The above analogy for different system sizes (for a fixed set of disorder and hopping parameters) along with those displayed in Figure 3 and Figure 6 (for different disorder and hopping parameters but at a fixed system size) lend further credence to our Λ parameter-based theoretical formulation of the SPEE measures, i.e., Equation (34) and Equation (44). Recalling that these equations are applicable for arbitrary $N_{A}$ with $N_{B}=N-N_{A}$, it is desirable to numerically establish that the Λ-based analogy extends to the case $N_{A}\neq N_{B}$ too. The collective response of the basis states of one subsystem to another also hints at the importance of relative subsystem sizes in the potential emergence of $\xi_{ent}$ and thereby for the finite size scaling of SPEE. Indeed the existence of such a length scale was reported in the study [4], which was based on a finite size scaling analysis of the von Neumann entropy for engineered bipartite Gaussian states (EPGSs). A finite size scaling of the average SPEE for the $N_{A}\neq N_{B}$ case was also reported in [11], with one of the subsystems consisting of a single site. A detailed analysis of the case $N_{A}\neq N_{B}$ and its impact on $\xi_{ent}$ is currently under progress and will be discussed in a subsequent work.

**Figure 8:**
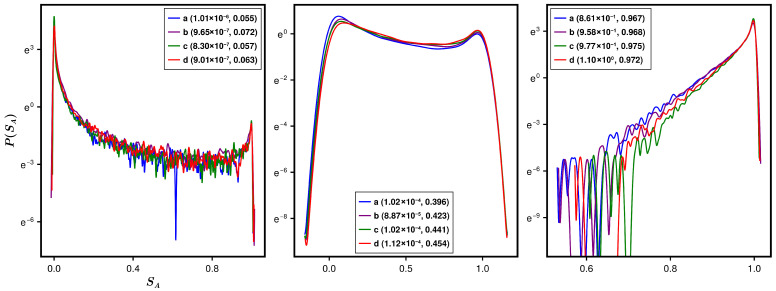
Distribution of SPEE for Anderson Hamiltonian. The figure illustrates the SPEE distribution for a typical eigenstate at energy E=0 of an Anderson Hamiltonian for four different combinations of systems parameters $w,w_{1},k$ for a fixed L=12. The combinations are chosen such that Equation (25) gives almost same Λ value for them. The results for three different Λ are displayed in three panels, with both $\left(\Lambda ,\langle S_{A}\rangle \right)$ mentioned in the legend and the labels (a), (b), (c), and (d), referring to different combinations of $w_{1}$ and *k*: (a) $k=1,w_{1}=0.0$, (b), $k=2,w_{1}=0.0$, (c) $k=1,w_{1}=1.0$, and (d) $k=2,w_{1}=1.0$. The large deviation tails for the SPEE distribution for small and intermediate Λ values are clearly visible here.

The numerically observed success of the Λ-formulation for the first two moments of the SPEE statistics encourages one to seek up to what order of moments it would persist. An appropriate route to answer this query is by directly analyzing the SPEE distribution over the ensemble ρ(H) of *M* Hamiltonians. Although our present theoretical analysis is confined to the average and variance, we numerically analyze the SPEE distribution for an Anderson state at E=0 and for many combinations of systems parameters $w,w_{1},k$. To improve the statistics, we consider a spectral averaging of $S_{A}$ over N/100 eigenfunctions in the neighbourhood of E=0. We recall that many different combinations of systems parameters can lead to the same Λ value (following from Equation (16) and Equation (25)) and thereby same SPEE statistics. To verify this in case of the SPEE distribution, we again consider four different sets of $w,w_{1},k$, which lead to approximately the same Λ; the results for three different Λ values are displayed in Figure 8. As can be seen from the figure, the distributions for different system parametric combinations almost collapse to one curve if they share approximately the same Λ value. (An exact matching, although desirable, requires a detailed analysis of the average localization length ξ and therefore transfer matrix analysis, which is time-consuming and will be pursued elsewhere.) The figure also reveals large deviation tails for the SPEE distribution for small and intermediate Λ values and appears similar to the one reported for the bipartite entanglement entropy of a typical state of the random-field Heisenberg model [6]. Indeed, the average entanglement entropy of an arbitrary bipartite quantum state with Gaussian-distributed components (with arbitrary variances and mean values) reported in [4,5] also displays a behavior visibly similar to that of the average SPEE discussed in the current work.

**RP Ensemble:** We next consider the Rosenzweig–Porter Hamiltonian, with its ensemble density described by Equation (9) with $\mu =cN^{\alpha }$, where *N* is the size of the matrix. The initial state of the ensemble is chosen as μ→∞, which corresponds to the localized dynamics of the eigenfunctions and thereby separable state of the two sub-lattices. We again consider the eigenstates at E=0 and calculate the SPEE statistics for different free parameters *c* and α. Equation (16) gives in this case

(51) $$Y-Y_{0}=-\frac{N-1}{2(N+1)\gamma }\ln|1-\frac{2\gamma }{1+\mu }|\approx -\frac{1}{2\gamma }\ln|1-\frac{2\gamma }{1+\mu }|.$$

Here again we set γ=1/2. The *spectral complexity parameter* $\Lambda_{e}$ can now be determined from Equation (25). We note that, in contrast to the AE case, the local mean level spacing $\Delta_{local}\left(e\right)$ at energy *e* is now given as $\Delta_{local}\left(e\right)=\Delta \left(e\right)={\left(R_{1}\left(e\right)\right)}^{-1}$ (explained in Section SVII of the Supplementary Material), with $R_{1}\left(e\right)$ as the ensemble-averaged spectral density at the energy *e* and Δ(e) as the mean level spacing.

**Figure 9:**
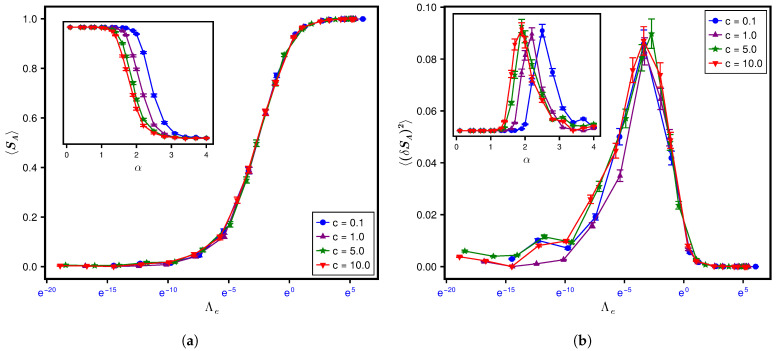
Evolution of SPEE measures for the RP ensemble. The figure illustrates the $\Lambda_{e}$-governed evolution of the (**a**) average and (**b**) variance of SPEE for the RP ensemble (with $\mu =cN^{\alpha }$, where *c* and α are the free parameters) for a fixed matrix size $N=12^{3}$. For comparison, the inset displays the α-governed evolution for different *c* values. As indicated by the collapse of curves already in terms of $\Lambda_{e}$, χ seems to be irrelevant here. This suggests an almost independence of χ from *c*, which thereby plays an insignificant role in the comparison of RPE with different *c* values. A consideration of χ is, however, relevant when compared with a different system, i.e., AE (see Figure 10). Here again the statistical uncertainties are small compared to symbol size, thus making error bars irrelevant.

The $\Lambda_{e}$ dependence of the average of $S_{A}$ and its variance over the ensemble ρ(H) is displayed in Figure 9, with the inset showing the corresponding dependence on α for different *c*. As evident from the figure, the statistics evolves along different paths for different *c* values, with α as the evolution parameter; it follows the same path, however, in terms of $\Lambda_{e}$, evolving rapidly from separable to the maximum limit. But again recalling that the evolution paths for different system parameters are expected to converge in terms of $\Lambda =\chi \Lambda_{e}$, this suggests an almost analogous χ-variation with $\Lambda_{e}$ for all *c* values considered in our analysis.

To gain insights into higher-order statistics for the RP case, here again we numerically analyze the SPEE distribution for the eigenstates chosen from the neighborhood of E=0 and for many c,α combinations. The results displayed in Figure 11 indicate, in contrast to the Anderson case, an absence of the large deviation tails for the RP case in the small Λ regime. The behavior in the other regimes for the two cases is, however, analogous. Indeed, such behavior is expected because the diffusion equation for the distribution is governed by a single parameter only in the large Λ limit. (With our primary focus on the average $S_{A}$ in the current work, the mathematical details for the diffusion equation are omitted here, but it can be derived following similar steps to those given in [5] for engineered bipartite Gaussian states (EPGSs).)

As mentioned near Equation (49), the analogy of Λ-governed evolution of SPEE measures is not only predicted for a specific Hamiltonian under different system conditions, but also expected to occur for two different Hamiltonians if both belong to same class of global constraints, e.g., symmetries and conservation laws ([15]). As the matrices in both AE and RP ensembles are real-symmetric, subjected to no additional constraints, the complexity constants for both systems can be chosen same (see the discussion in Section SV of the Supplementary Material). This suggests that, under system conditions leading to $\Lambda_{ae}\left(e\right)=\Lambda_{rp}\left(e\right)$, the SPEE statistics for AE and RP should be analogous. In addition, the Λ-governed paths of their SPEE evolution are theoretically expected to be analogous too. Considering same *N* for both systems, Equation (26) gives $\frac{\chi_{rp}}{\chi_{ae}}=\frac{\kappa_{ae}^{2}{\langle I_{2}\rangle }_{ae}^{2}}{\kappa_{rp}^{2}{\langle I_{2}\rangle }_{rp}^{2}}$ and thereby $\Lambda_{e,rp}$∼$\frac{\kappa_{ae}^{2}}{\kappa_{rp}^{2}}\frac{{\langle I_{2}\rangle }_{ae}^{2}}{{\langle I_{2}\rangle }_{rp}^{2}}\Lambda_{e,ae}$ (with subscripts “ae” and “rp” implying the measures for AE and RP, respectively). Here again due to lack of knowledge about the constants $\kappa_{rp}$ and $\kappa_{ae}$, we determine them by a numerical estimation. (The latter is based on first noting, from Figure 3 and Figure 9, the values of $\Lambda_{e,ae}$ and $\Lambda_{e,rp}$ that correspond to ${\langle S_{A}\rangle }_{rp}\approx {\langle S_{A}\rangle }_{ae}$. With the latter equality predicted to occur at $\Lambda_{ae}=\Lambda_{rp}$, this implies the relation $\frac{\chi_{rp}}{\chi_{ae}}=\frac{\Lambda_{e,ae}}{\Lambda_{e,rp}}$, which in turn gives $\frac{\kappa_{ae}^{2}}{\kappa_{rp}^{2}}=\frac{{\langle I_{2}\rangle }_{rp}^{2}\Lambda_{e,rp}}{{\langle I_{2}\rangle }_{ae}^{2}\Lambda_{e,ae}}$. Substitution of numerically determined values of $\langle I_{2}\rangle$ and $\Lambda_{e}$ for both cases now gives the desired ratio.)

**Figure 10:**
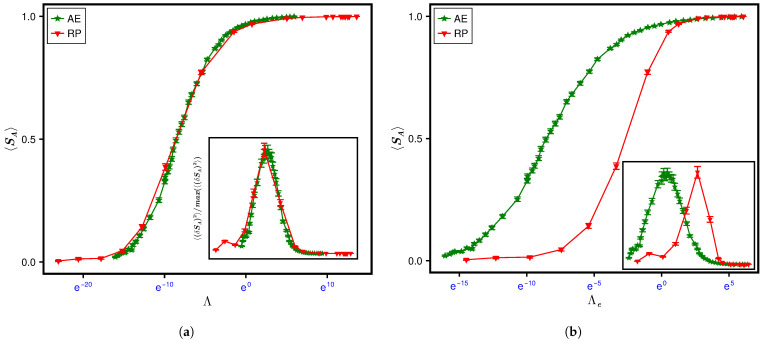
Comparison of SPEE measure evolution for the RP and Anderson ensemble. The comparison of the evolution of the average SPEE for one of the cases for the RP (c=0.1) and Anderson ensemble (AE) ($k=2,w_{1}=0.0$) is shown. The panel (**a**) displays the evolution in terms of Λ and (**b**) in terms of $\Lambda_{e}$. The insets show a similar comparison for the normalized variance of the SPEE. A clear deviation in the bottom panel indicates the relevance of χ for the SPEE statistics.

**Figure 11:**
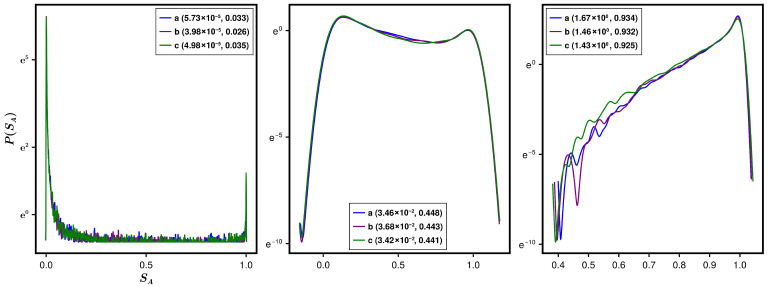
Distribution of SPEE for the RP ensemble. The SPEE distribution for a typical eigenstate at E=0 of the RP ensemble for three different *c* values at a fixed $N=12^{3}$ is shown. For different *c* but fixed $\Lambda_{e}$, the distributions tend to collapse almost to a single curve. The labels (a), (b), and (c) in the legend refer to the $\Lambda_{e}$ and $\langle S_{A}\rangle$ values for the cases c=0.1,1, and 10, respectively. Notwithstanding the $\Lambda_{e}$ value being only nearly equal, the curves for different *c* values collapse onto a single curve. We note that the y-axes in the center and the rightmost panels are on a log scale, but the one in the leftmost panel is chosen to be on a pseudo-log scale; the latter is required to visualize the narrow distribution and vanishing probability for $S_{A}>0$ in the small $\Lambda_{e}$ regime. This is in contrast to the Anderson case (Figure 8), with large deviation tails for small $\Lambda_{e}$, although the distributions in the intermediate and large $\Lambda_{e}$ regimes for the RP and AE cases show similar trends.

Figure 10 displays a comparison of $\langle S_{A}\rangle$ statistics for RP ensemble and Anderson ensemble for one set of their system parameters (sufficient as collapse for different sets is already displayed); the good agreement of the two curves in top panel, with Λ as the independent variable, and its absence in lower panel, with $\Lambda_{e}$ as the independent variable, once again emphasizes the role of Λ as the relevant single parameter for SPEE-statistics (instead of $\Lambda_{e}$). Based on the above analogy, a finite size scaling behavior for the SPEE statistics is expected in the RP case too; this analysis is currently under progress.

The Λ-based analogy depicted in Figure 10 corresponds to the eigenstates chosen near energy E=0 for both AE and RP ensembles. But, as mentioned near Equation (49), the analogy need not be confined to E=0 only and is theoretically predicted for other energies too (i.e., the two systems at the same or different energies) if the condition in Equation (49) is satisfied. A numerical analysis of the above prediction is currently being pursued in our follow-up work on many-body entanglement and we hope to report it in the near future. (Meanwhile we note that a detailed theoretical as well as numerical verification of Λ-based universality for the spectral statistics at different energies of chiral Hamiltonians is discussed in [27].)

## 7. Conclusions

In this work, we have analyzed the SPEE dynamics in disordered Hamiltonians as the system conditions vary. The theoretical approach presented here is based on the complexity parameter formulation of the eigenfunction statistics of the Hamiltonians represented by multiparametric Gaussian ensembles; the formulation was developed and investigated in detail in [15] based on an exact diagonalization of the diffusion equation for the ensemble density. Hoping to extend the potential applicability of our formulation to non-Gaussian cases too, we have also included here an alternative derivation based on second-order perturbation theory of quantum Hamiltonians and the Markovian dynamics assumption.

Our theory predicts the existence of a common mathematical formulation of the SPEE for the typical eigenstates of a wide range of single-particle Hamiltonians, represented by different multivariate Gaussian ensembles but subjected to the same symmetry constraints and conservation laws. As the ensemble parameters contain information about the system details and are therefore different for ensembles representing different Hamiltonians, the system dependence appears in the formulation collectively through a single parameter Λ, a functional of the system parameters. This in turn implies an analogy of the SPEE statistics for different quantum states (same or different Hamiltonians) if they share same Λ values and thereby reveals a great deal of universality hidden underneath the world of quantum states. While our SPEE formulation is based on an exact theoretical formulation for the JPDF of the eigenfunction components, the robustness of our claim renders numerical verification desirable. In particular, we have tested out theoretical predictions for many system conditions of two prototypical Hamiltonians, viz., the 3D Anderson model and the Rosenzweig–Porter ensemble. A minor drawback of our analysis at this stage is the approximate information for χ; while crucial to determine Λ, this, however, is a challenging technical problem due to eigenvalue–eigenfunction correlations, with insights seemingly possible only through numerical analysis. We recall that the spectral complexity parameter $\Lambda_{e}$ is related to Λ through χ. As discussed in detail in [15,22], $\Lambda_{e}$ plays an important role in determining the critical spectral statistics and the multifractal exponents, relevant criteria for the localization-to-delocalization transitions in complex systems. The $\Lambda_{e}$ dependence of the SPEE statistics (through Λ) can then be used to study the latter’s connection to multifractality exponents; we hope to pursue this study in future.

Our SPEE analysis presented above reveals a potentially useful insight about the entanglement entropy in quantum systems. The prevailing notion in the research community about the SPEE is that it is at best a measure of the quantum correlations with classical counterparts and does not have as mysterious an origin as the bipartite entanglement entropy, which characterizes the correlations with no classical counterpart. A comparison of our SPEE results with those for bipartite entanglement entropy reported in [4,5,28], however, indicates a similar dependence on the complexity parameter (the studies [4,5,28] were based on the bipartite quantum states described by multivariate Gaussian ensembles). A similar dependence is also revealed by our current study of the bipartite entanglement entropy of many-body states; we expect to report it in the near future. This is not surprising: it has been known for last few decades that the complexity, irrespective of its diverse origin, often leads to independence from the system specifics. In addition, our preliminary analysis of the finite size scaling of SPEE measures suggests the existence of a new length scale relevant for separability to the single-particle entanglement transition. With the latter expected to be different from the average localization length that governs the typical localization-to-delocalization transition of the eigenstates, the information may potentially lead to new insights not only into the quantum dynamics of single-particle Hamiltonians but for many-body cases too.

A common mathematical formulation for the entanglement dynamics in single-particle systems has by itself many implications. The first and foremost among them is the existence of an infinite range of universality classes of the SPEE statistics for finite systems, characterized by the *strength complexity parameter* Λ, with the latter taking continuous values between 0 and ∞. For infinite system sizes, the size dependence of Λ, however, results in its rapid approach either to 0 or ∞. If the subsystems are initially chosen to be separable, the SPEE either approaches the separability limit or the maximum entanglement limit. If, however, a subtle conspiracy of the system conditions can lead to size-independent Λ for an arbitrary system size, or a non-zero, finite Λ even in an infinite size limit, the statistics is then predicted to be different from both limits. In general, in an infinite size limit, only discrete values of non-zero, finite Λ are possible; this implies only a partial entanglement of the two subsystems at the energy under consideration. Statistically, however, this entanglement behavior need not be unique and may be achieved by a change in system conditions as well as energy for a fixed pair of subsystems or a change of subsystems with or without other changes. Alternatively stated, the universality implied by the Λ formulation reveals the lack of sensitivity of the SPEE to specific system details; it depends only on their collective information. A potential extension of this formulation for multi-particle systems is also important and thus desirable. The entanglement can be then studied in the traditional sense, where a partition consists of a fraction of the total number of particles. An interesting path to pursue will be to develop a single-parametric formulation of the entanglement dynamics for such cases and thereby seek the universality classes among bipartite and multipartite entanglement. This work is currently in progress.

## Data Availability

The code is available on the following repository: https://github.com/shekhardevanshu/Anderson.

## Supplementary Materials

The following supporting information can be downloaded at https://www.mdpi.com/article/10.3390/e28010029/s1, Figure S1: Comparison of inverse participation ratio $I_{2}$ for an eigenfunction at e=0 for two different hopping ranges of the Anderson Hamiltonian (matrix size $N=12^{3}$ and for a fixed off-diagonal disorder $w_{1}=1.0$). A variation of diagonal disorder *w* leads to change in $Y-Y_{0}$ and $I_{2}$. The label k=1 and k=2 correspond to nearest and next-nearest neighbour hopping, respectively. Here s=0.02 for k=1 and s=0.0 for k=2.

## Appendix A. Definition of Symbols

**Table A1:** The table given below gives a brief description of the main symbols used in this paper. Other symbols are defined in the text where they are introduced for the first time.

*d*	Dimension of the system represented by Hamiltonian *H*
ρ(H)	JPDF of all matrix elements of *H*
$v_{kl},b_{kl}$	Variance and mean, respectively, of matrix element $H_{kl}$
$\rho_{1}\left(H\right)$	JPDF of matrix elements of Anderson Hamiltonian
$w,w_{1},t$	Free system parameters of Anderson Hamiltonian
$\rho_{2}\left(H\right)$	JPDF of matrix elements of Rosenzweig–Porter Hamiltonian
μ,c,α	Free system parameters of Rosenzweig–Porter Hamiltonian
〈X〉	Ensemble average of an arbitrary variable *X*
*Y*	Ensemble complexity parameter defined in Equation (16)
$P_{ef,ev}$	JPDF of all eigenfunctions and eigenvalues of *H*
$P_{\psi }$	JPDF of all components of an eigenfunction ψ
$\psi_{n}$	$n^{th}$ component of ψ in the chosen basis
ξ(e)	Ensemble averaged localization length at energy *e*
$\xi_{ent}$	Length scale related to separability-to-maximum entanglement transition
$\langle I_{2}\left(e\right)\rangle$	Ensemble-averaged inverse participation ratio at energy *e*
$\Delta_{e}\left(e\right)$	Mean level spacing at energy *e*
$\Delta_{local}\left(e\right)$	Local mean level spacing
$\Lambda_{e}$	Spectral complexity parameter defined in Equation (25)
Λ	$=\chi \Lambda_{e}$ with χ defined in Equation (26)
$S_{A}$	Single-particle entanglement entropy of ψ
$\langle S_{A}\rangle ,\langle \delta S_{A}^{2}\rangle$	Ensemble average and variance of $S_{A}$

## Appendix B. Goodness of Fit

The measure $R^{2}$ for goodness of fit is defined as follows:

(A1) $$\begin{array}{c} R^{2}\equiv 1-\frac{\sum_{i=1}^{n}{\left(y_{i}-{\hat{y}}_{i}\right)}^{2}}{\sum_{i=1}^{n}{\left(y_{i}-\bar{y}\right)}^{2}}, \end{array}$$

where $\bar{y}=\frac{1}{n}\sum_{i=1}^{n}y_{i}$, with $y_{i}$ denoting the numerically obtained values, ${\hat{y}}_{i}$ the predictions from the conjecture, and *n* the total number of data points.
