# Supplementary material for: Single-Particle Entanglement Dynamics in Complex Systems
Source: Entropy (Basel). 2025 Dec 25;28(1):29. doi: 10.3390/e28010029 (PMC12839925; doi:10.3390/e28010029)
Supplement: Supplementary file 1 [file entropy-28-00029-s001.zip › entropy-3953613-supplementary.pdf]

entropy

MDPI

Article

Supplementary File

Devanshu Shekhar and Pragya Shukla *

Department of Physics, Indian Institute of Technology, Kharagpur-721302, West Bengal, India
* Correspondence: shukla@phy.iitkgp.ac.in

Abstract

Here we explain the detailed steps for the derivation of equations used in the main text and also include some theoretical explanations and figures, not included in main text to avoid defocusing of the text.

1. Complexity Parameter Formulation of the ensemble density: derivation of Equation (12) and Equation (17) of main text

We consider an ensemble of $N \times N$ Hermitian matrices H with real-symmetric elements H_{kl} distributed as Gaussians with arbitrary mean and variances; the four linearly independent components of each matrix element are chosen to be statistically independent. A general form of the probability density $\rho(H) \equiv \prod_{k,l;k \le l} \rho_{kl}(H_{kl})$ of the ensemble can then be given by

$$\rho(H, h, b) = C\exp\left[- \sum_{k \le l} \frac{(H_{kl} - b_{kl})^2}{2v_{kl}} \right] \tag{S1}$$

with v as the set of the variances $v_{kl} =< H^2_{kl} >$ and b as the set of all mean values $< H_{kl} >= b_{kl}$. As obvious, in the limit $v_{kl} \to 0$, Equation (2) corresponds to the non-random nature of H_{kl} (that is, $\rho_{kl}(H_{kl}) = \delta(H_{kl} - b_{kl})$).

As the physical properties of a Hermitian operator e.g. Hamiltonian can in principle be derived from its eigenvalues and eigenfunctions, it is necessary to determine the joint probability distribution of its eigenvalues and eigenfunctions. This however requires a transformation of $\rho(H)$ from the matrix space to eigenvalue and eigenfunction space and thereafter integrating over undesirable variables e.g. the joint probability distribution of the eigenvalues requires an integration over eigenvector space which is well-known to be technically challenging. In addition, even if one is able to derive the desired distribution using some approximations, those are applicable only for a given parametric range and need not be applicable once the system conditions change. This motivates us to abandon the integration route and seek a route that is applicable for a wide range of parametric conditions. This can be described as follows.

Consider a physical system described by a Hamiltonian H. The underlying complexity (e.g. many body interactions, disorder etc.) of the system renders it difficult to exactly determine the matrix elements of H in a physically relevant fixed basis space (e.g. the one which preserves its underlying symmetries and conservation laws). The approximate information to determine matrix H then leaves its statistical description as the only option and it can be best described by an ensemble say $\rho(H)$. For the latter to be an appropriate description of the system, the ensemble parameters must be determined from the system parameters. The latter can however change and this not only subjects the matrix H to change

Check for updates

Academic Editors: Michael Parker, Chris Jeynes, Stuart Walker and Luisberis Velazquez

Received: 13 October 2025
Revised: 18 December 2025
Accepted: 19 December 2025
Published: 25 December 2025

Copyright: © 2025 by the authors. Licensee MDPI, Basel, Switzerland. This article is an open access article distributed under the terms and conditions of the Creative Commons Attribution (CC BY) license.

as $H \to H + \delta H$, the ensemble parameters can also change. These changes however occur in two different spaces, with H_{kl} changing in matrix space and v_{kl}, b_{kl} changing in parameter space. It is therefore important to query whether the change $\rho(H, v, b) \to \rho(H + \delta H, v, b)$ can exactly be mimicked by the change $\rho(H, v, b) \to \rho(H, v + \delta v, b + \delta b)$ and how long that analogy persists? Alternatively, does the same ensemble keep representing the dynamics of the system in both matrix space as well as in parameter space? Indeed, using Gaussian nature of ρ, it is easy to verify that under a change of the parameters $v_{kl} \to v_{kl} + \delta v_{kl}$ and $b_{kl} \to b_{kl} + \delta b_{kl}$, the matrix elements H_{kl} undergo a diffusion dynamics along with a finite drift,

$$T\rho = L\rho \tag{S2}$$

where

$$\begin{aligned} T &\equiv \sum_{j=1}^{M} \frac{\partial}{\partial f_j} \\ L &\equiv \sum_{kl} \frac{\partial}{\partial H_{kl}} \left[\frac{g_{kl}}{2} \frac{\partial}{\partial H_{kl}} + \gamma H_{kl} \right] \end{aligned} \tag{S3}$$

with

$$\begin{aligned} f_j &= (1/2)\log|x_{kl}| + c_j \qquad \text{for } j = 1 \to M_1, \\ f_j &= \log|b_{kl}| + C_j \qquad \text{for } j > M_1 \end{aligned} \tag{S4}$$

with $x_{kl} \equiv 1 - \gamma\, \tilde{g}_{kl}\, v_{kl}$, $\tilde{g}_{kl} = 2 - \delta_{kl}$ and $g_{kl} = 1 + \delta_{kl}$. Here M corresponds to total number of ensemble parameters participating in evolution, with M_1 and $M - M_1$ as the number of non-zero parameters x_{kl} and b_{kl}, respectively. The γ is an arbitrary parameter, giving the variance of the matrix elements at the end of the evolution [1,2].

An important point worth emphasizing here is as follows. We begin with the ensemble density Equation (S1) with no information about its past i.e how the system arrives at this state. In contrast, the differential equation (S2) now describes as to what happens if the dependent variables are subjected to a small change i.e $H_{kl} \to H_{kl} + \delta H_{kl}$, $v_{kl} \to v_{kl} + \delta v_{kl}$ and $b_{kl} \to b_{kl} + \delta b_{kl}$ and thereby introduces a dependence on the initial condition and its solution can be given as $\rho(H, v, b|H_0, v_0, b_0)$. Thus the dynamics generated by the differential operator L describes a flow of the ensemble density $\rho(H, v, b|H_0, v_0, b_0)$ in real-symmetric matrix space, from an arbitrary initial real-symmetric matrix H_0 due to a change in matrix elements H_{kl}. In contrast the parametric operator T generate a multi parametric flow of the ensemble density $\rho(H, v, b|H_0, v_0, b_0)$ in the ensemble parameter space $\{v, b\}$, from an arbitrary initial ensemble $\rho_0(H_0, v_0, b_0)$.

As discussed in detail in [1,2], it is possible to map the dynamics of $\rho(H; v, b)$ in v, b parameter space to another parametric space $t \equiv \{t_1, \ldots, t_M\}$, referred as the complexity parameter space, in which $\rho(H; t)$ undergoes a single parametric evolution, with t_1 as the only evolution parameter:

$$\frac{\partial \rho}{\partial t_1} = L\rho_1, \qquad \frac{\partial \rho}{\partial t_\alpha} = 0 \quad \forall\, \alpha > 1, \tag{S5}$$

The parameters $t_1, \ldots, t_M$ for each one of the above cases can be obtained by solving the characteristic set of equations

$$\frac{\mathrm{d}f_1}{f_1} = \frac{\mathrm{d}f_2}{f_2} = \ldots = \frac{\mathrm{d}f_M}{f_M} = \frac{\mathrm{d}t_\alpha}{\delta_{\alpha 1}} \tag{S6}$$

A particular solution of the above equation can be given as, with $\mu \equiv kl$,

$$t_1 = \sum_{\mu=1}^{M} q_{\mu;1} \ln |f_\mu| + c_0, \tag{S7}$$

$$t_\alpha = \sum_{\mu} q_{\mu;\alpha} \log |f_\mu|, \qquad \alpha > 1 \tag{S8}$$

with $q_{\mu;\alpha}$ as arbitrary constants subjected to condition $\sum q_{\mu;\alpha} = \delta_{\alpha 1}$. (This can be checked by a direct substitution in relation $\sum_\mu \frac{\partial t_\alpha}{\partial f_\mu} = \delta_{\alpha 1}$). While many solutions satisfying the above condition are possible, the appropriate solution is the one that is also applicable for initial ensemble (as the constants of evolution are constants for initial ensemble too). Indeed the choice of constants $q_{\mu;1}$ in Equation (S7) depends on the evolving ensemble parameters. For example, if one of the f_μ remains zero throughout the evolution and does not participate in the evolution, the corresponding coefficient $q_{\mu;1}$ can be set to zero. For the case in which all f_μ remain non-zero throughout the evolution, it is appropriate to choose $q_{\mu;1} = 1/M$ (for all μ). The choice fulfills the condition $\sum_{\mu=1}^{M} q_{\mu;1} = 1$ and t_1 can be given as

$$t_1 = -\frac{1}{\gamma M} \ln \left[\prod_{k \le l}{}' \prod_{s=1}^{\beta} |x_{kl}| \quad |b_{kl}|^2 \right] + C \tag{S9}$$

here $\prod'$ implies a product over non-zero b_{kl} and x_{kl}. Further, C is a constant determined by the initial distribution. Being a function of various distribution parameters h_{kl} and b_{kl}, t_1 can be referred as the complexity parameter. As t_1 acts as the evolution parameter while $t_2, \ldots, t_M$ are the constants of evolution, we refer t_1 as Y to emphasize the difference.

The evolution reaches a steady state when $\partial\rho/\partial Y \to 0$ with the ensemble $\rho(H)$ approaching the Wigner-Dyson limit, $\rho \propto e^{-(1/2)\mathrm{Tr}H^2}$. The flow described by Equation (S5) can start from any initial state; the only constraint on the choice is that the parameters t_j for the initial ensemble should be same as those for the ensemble $\rho(H, y, b)$.

As the complexity constants $t_2, \ldots, t_M$ do not appear in Equation (S5), its solution and therefore the ensemble-statistics is same even for different sets of complexity constants. Alternatively stated, the distribution $\rho(H|H_0)$, depends on the multiple parameters v_{kl}, b_{kl} (for all k, l) only through a function Y. Indeed a solution of Equation (S5) can be given as

$$\rho(H, Y|H_0, Y_0) \equiv \rho(H, Y, t_2, .., t_M|H_0, Y_0, t_2, .., t_M) \propto \exp\left[-\gamma \frac{\mathrm{tr}(H - \eta H_0)^2}{2(1 - \eta^2)} \right] \tag{S10}$$

where $\eta = e^{-(Y - Y_0)}$. An integration over initial probability density now leads to

$$\rho(H, Y, t_2, .., t_M) = \int \rho(H, Y|H_0, Y_0)\, \rho(H_0, Y_0, t_2, .., t_M) \mathrm{d}H_0. \tag{S11}$$

The above in turn describes the mapping of $\rho(H, v, b)$ in Equation (S1) to the complexity parameter space $\rho(H, Y, t_2, .., t_M)$ and no longer depends on any initial condition.

The common mathematical framework suggests an analogous formulation for physical properties, and their classification into universality classes defined by the single (complexity) parameter Y.

2. First and second moments of eigenvalues and eigenfunctions: derivation of Equation (19) of main text

Consider an $N \times N$ Hermitian matrix H with Y as a parameter, U as the $N \times N$ eigenvector matrix of $H(Y)$, (an orthogonal matrix satisfying $O^T O = 1$) and E as the $N \times N$

diagonal matrix of its eigenvalues, $E_{mn} = e_n\ \delta_{mn}$. A small perturbation of H changes its eigenvalues and eigenfunctions. Using standard perturbation theory for Hermitian operators and by considering matrix $H + \delta H$ in the eigenfunction representation of matrix H, the second order change in the jth component O_{jn} of an eigenfunction O_n, in an arbitrary basis $|j\rangle$, $j = 1, \ldots, N$, due to a small change δY can be described as

$$\delta O_{jn} = \sum_{m \neq n} \frac{\delta H_{mn}}{e_n - e_m} H_{jm} + \sum_{m,m' \neq n}^{N} \frac{\delta H_{mn}\ \delta H_{m'n}}{(e_n - e_m)(e_n - e_{m'})} O_{jm} - \sum_{m \neq n}^{N} \frac{\delta H_{mn}\ \delta H_{nn}}{(e_n - e_m)^2}\ O_{jm} - \frac{1}{2} O_{jn} \sum_{m \neq n}^{N} \frac{\delta H_{mn}\ \delta H_{nm}}{(e_n - e_m)^2} \tag{S12}$$

Similarly, a small change δe_n in the eigenvalue e_n can be given as

$$\delta e_n = \delta H_{nn} + \sum_{m \neq n} \frac{|\delta H_{mn}|^2}{e_n - e_m} + o((\delta H_{mn})^3) \tag{S13}$$

where $H_{mn} = e_n\ \delta_{mn}$ at value Y of complexity parameter (due to $H + \delta H$ being considered in the diagonal representation of H).

As given. in Equation (S5), the $Y \equiv t_1$ governed evolution of $\rho(H)$ can be described as

$$\frac{\partial \rho}{\partial Y} = \sum_{\mu,\nu} \frac{\partial}{\partial H_{\mu\nu;s}} \left[\frac{g_{\mu\nu}}{2} \frac{\partial}{\partial H_{\mu\nu}} + H_{\mu\nu} \right] \tag{S14}$$

where with $g_{\mu\nu} = 2$ or 1 for $\mu = \nu$ and $\mu \neq \nu$, respectively,

In general, assuming Markovian process, the parametric diffusion of the joint probability distribution $P_x(x_1, \ldots, x_N; Y)$ of N variables x_n, $n = 1, \ldots, N$ from an arbitrary initial condition, with Y as the parameter, is given by the standard Fokker-Planck approach,

$$\frac{\partial P_x}{\partial Y}\ \delta Y = \frac{1}{2} \sum_{k,l=1}^{N} \frac{\partial^2}{\partial x_k \partial x_l} \left(\langle \delta x_k \delta x_l \rangle\ P_x \right) - \sum_{k=1}^{N} \frac{\partial}{\partial x_k} \left(\langle \delta x_k \rangle\ P_x \right) \tag{S15}$$

A comparison of Equation (S15) with the above equation thereby gives the relevant information for the moments of $\rho(H)$,

$$\langle \delta H_{\mu\nu} \rangle = -\gamma\ H_{\mu\nu}\ \delta Y, \tag{S16}$$

$$\langle (\delta H_{\mu\nu})^2 \rangle = g_{\mu\nu}\ \delta Y, \qquad \langle \delta H_{\mu\nu}\ \delta H_{\mu'\nu'} \rangle = 0 \tag{S17}$$

with $g_{\mu\mu} = 2$ and $g_{\mu\nu} = 1$ for $\mu \neq \nu$. All other averages are of a higher order in δY and can be ignored for a small change in Y.

An ensemble average of Equation (S12) would lead to moments for the eigenfunction components. This however requires information about the moments of $\langle \delta H_{mn}^2 \rangle$ as well as $\langle \delta H_{mn} O_{jm} \rangle$ and $\langle \delta H_{mn} \delta H_{kn} O_{jn} \rangle$. A calculation of these averages is easier if each O_n is represented in a basis $|j\rangle$ in which the perturbation applied to $H(Y)$ is random; this renders the elements of δH matrix statistically independent of the components O_{jn}. (Also note the ensemble averaging is over the ensemble of δH matrices for a fixed H at Y). An appropriate choice for $|j\rangle$ for this purpose is the eigenfunction basis of H_0 at $Y = Y_0 = 0$. Now using Equations (S16–S17), it is easy to see that the ensemble averaged O_{jn} has a non zero contribution only from the last term of Equation (S12):

$$\langle \delta O_{jn} \rangle = -v^2 \sum_{m=1,m\neq n}^{N} \frac{O_{jn}\, \delta Y}{(e_n - e_m)^2} \tag{S18}$$

with angular brackets implying conditional ensemble averages with fixed e_n, O_n, $n = 1, \ldots, N$ and $\beta = 1$ or 2 for H as a real-symmetric or complex Hermitian matrix. But the 2nd moment of the eigenvector components has a contribution only from the first term in Equation (S12) (up to first order in δY),

$$\langle \delta O_{jn}\, \delta O_{kl} \rangle = 2\, v^2 \left(\sum_{m=1,m\neq n}^{N} \frac{O_{jm}\, O_{km}\, \delta_{nl}}{(e_n - e_m)^2} - \frac{O_{jl}\, O_{kn}\, (1 - \delta_{nl})}{(e_n - e_l)^2} \right) \delta Y \tag{S19}$$

Similarly an ensemble average of Equation (S13) gives, up to first order of δY,

$$\begin{aligned} \langle \delta e_n \rangle &= 2\, v^2 \left[N_A - \frac{\gamma}{\beta\, v^2}\, e_n + \sum_{m=1,m\neq n}^{N} \frac{1}{e_n - e_m} \right] \delta Y \\ \langle \delta e_n \delta e_m \rangle &= 8\, v^2\, e_n\, \delta_{nm}\, \delta Y \end{aligned} \tag{S20}$$

Further, to first order in δY, the ensemble averaged correlation between δe_k and δO_{jn} is zero (for both $\beta = 1$ or 2),

$$\langle \delta e_k\, \delta O_{jn} \rangle = -2\, v^2 \sum_{m=1,m\neq n}^{N} \frac{H_{mn}}{(e_n - e_m)}\, O_{jn}\, \delta Y = 0 \tag{S21}$$

Relevant information from the moments of eigenvalues and eigenfunction components of H can now be derived by using standard Fokker-Planck approach (Equation (S15)). As, for finite Y, the moments for the eigenfunction components depend on the eigenvalues too, we first write the diffusion equation for the joint probability density $P_{ef,ev}(\{O_n\}, \{e_n\}; Y)$ at perturbation strength Y where $\{O_n\}$ and $\{e_n\}$ refer to the sets of all eigenvectors $O_1, \ldots, O_N$ and eigenvalues $e_1, e_2, .., e_N$:

$$\frac{\partial P_{ef,ev}}{\partial Y} = (\mathcal{L}_U + \mathcal{L}_E) P_{ef,ev} \tag{S22}$$

where $\mathcal{L}_U$ and $\mathcal{L}_E$ refer to two parts of the Fokker-Planck operator corresponding to eigenvalues and eigenfunction components, respectively. Here $\mathcal{L}_U$ is given as

$$\mathcal{L}_U\, \delta Y = \sum_{j,n=1}^{N} \frac{\partial}{\partial O_{jn}} \left[\frac{1}{2} \sum_{k,l=1}^{N} \left(\frac{\partial}{\partial O_{kl}} \langle \delta O_{jn} \delta O_{kl} \rangle - \langle \delta O_{jn} \rangle \right) \right] \tag{S23}$$

and $\mathcal{L}_E$ is

$$\mathcal{L}_E\, \delta Y = \sum_n \frac{\partial}{\partial e_n} \left[\frac{1}{2}\, \frac{\partial}{\partial e_n} \langle (\delta e_n)^2 \rangle - \langle \delta e_n \rangle \right] \tag{S24}$$

Note here $P_{ef,ev}$ is subjected to following boundary condition: $P_{ef,ev} \to 0$ for $O_{jn} \to \pm\infty, \lambda_n \to (-\infty, \infty)$ for $j, n = 1 \to N$; this follows because the higher order moments of the ensemble density are assumed to be negligible.

A substitution of the moments (Equations (S18, S19, S20, S21)) in Equation (S22) followed by latter's integration over all undesired variables will then lead to an evolution equation for the joint probability density of the desired combination of eigenfunctions and eigenvalues.

3. Joint distribution of all components of an eigenfunction: derivation of Equation (24) of main text

Equation (S22) describes the complexity parameter governed evolution of the joint probability density function $P_{ef,ev}(O_1, \ldots, O_N; e_1, \ldots, e_N)$. Relevant information for the distribution of the components of an arbitrary eigenfunction of H can now be derived as follows.

The JPDF of the components O_{nk}, of an eigenstate, say O_k of H lying between ψ_n and $\psi_n + \mathrm{d}\psi_n$ with $n = 1 \to N$, can be given as

$$P_\psi(\psi_1, ..., \psi_N; Y) = \int \delta_{\psi,k}\, P_{ef,ev}\, \mathrm{D}\Omega \tag{S25}$$

where $\mathrm{D}\Omega \equiv \prod_{j=1}^N \mathrm{D}e_j \prod_{j=1}^N \mathrm{D}O_j$ is the volume element in the eigenvalue-eigenvector space and

$$\delta_{\psi,k} = \delta(\Psi - O_k) \equiv \prod_{n=1}^N \delta(\psi_n - O_{nk}) \tag{S26}$$

Partial differentiation of Equation (S25) with respect to Y, subsequent substitution of Equation (S22) and repeated partially integration leads to the diffusion equation for P_ψ:

$$\frac{\partial P_\psi}{\partial Y} = \frac{1}{4}\left[\sum_{m=1}^N \frac{\partial}{\partial \psi_m}\left[\psi_m\, Q^{02}_{mm;k}\right] + \sum_{m,n=1}^N \frac{\partial^2}{\partial \psi_m \partial \psi_n} Q^{12}_{mn;k}\right], \tag{S27}$$

Here the terms obtained by partial integration and evaluated at the integration limits contribute to zero (assuming vanishing of the probability as the components ψ_k approach $\pm\infty$). with

$$Q^{rs}_{mn;k} = \sum_{j;j\neq k} \int \delta_{\psi,k} \frac{(O_{mj}O_{nj})^r}{(e_k - e_j)^s} P_{ef,ev}\, \mathrm{D}\Omega, \tag{S28}$$

$$Q^{rs}_{mn;k} = \sum_{j;j\neq k} \int \frac{(O_{mj}O_{nj})^r}{|e_k - e_j|^s} P_2\, \mathrm{d}e_j\, \mathrm{d}e_k\, \mathrm{D}O_j. \tag{S29}$$

Here $P_2 = P_2(\Psi, \Phi, e, e')$ is the joint probability density of all the components of two eigenvectors Ψ and Φ along with their eigenvalues e and e', respectively:

$$P_2 = \int \delta_{\psi,k}\, \delta_{\phi,j}\, P_{ef,ev} \prod_{l=1}^N \mathrm{d}e_l\, \mathrm{D}O_l \tag{S30}$$

where $\delta_{\psi,k}$ is defined in Equation (S26). Note

$$P_\Psi(\Psi, e) = \int P_2\, \mathrm{d}e'\, \mathrm{D}\Phi \tag{S31}$$

As clear from Equation (S27), its right side contains functions which are not explicitly written in terms of P_ψ. In [3], Equation (S28) was approximated as

$$Q^{rs}_{mn;k} \approx \frac{(N-1)^{1-r}}{\Delta^s_{local}} \, (\delta_{mn} - \psi_m \psi_n)^r \, P_\Psi \tag{S32}$$

with $\Delta_{local}(e)$ as the *local* mean level spacing at energy $e = e_k$ (see Equation (22) of [3]), ($\Delta_{local} = \frac{N\Delta_e}{\xi^d}$ for a d dimensional system with average localization length ξ and Δ_e as the mean level spacing at the energy e.) The approximation in Equation (S32) was however based on an assumed weak statistical correlation between the eigenvalues and the eigenfunctions. Here we consider its improvement to include more generic regimes, based on the following ideas: (i) the eigenvalues at a distance more than few mean level spacing are uncorrelated, (ii) the average correlation between components of an eigenfunction is almost same as another eigenfunction if their eigenvalues are approximately equal. Using these ideas, we now have (derivation given in section IV)

$$Q^{02}_{mn;k} \approx \left(\frac{2}{\Omega_e}\right)^2 (N-1) \;\; P_\Psi(\Psi, e), \tag{S33}$$

$$Q^{12}_{mn;k} \approx \left(\frac{2}{\Omega_e}\right)^2 (\delta_{mn} - \psi_m \psi_n) \;\; P_\Psi(\Psi, e) \tag{S34}$$

where Ω_e, a function of both $Y - Y_0$ as well as e, is an important system-specific spectral-range defined as follows: the eigenvalues at distances more than Ω_e around e, are uncorrelated. In general, it is of the order of few local mean level-spacings: $\Omega_e = N_k \, \Delta_{local}$. Here N_k is the number of eigenvalues in the range, intuitively expected to be related to the inverse participation ratio $I_2 = \frac{1}{N} \sum_{n=1}^{N} |\psi_n|^4$ at energy e i.e. $N_k \sim N \, \langle I_2(e) \rangle$. This suggests

$$\Omega_e \approx N \, \langle I_2(e) \rangle \, \Delta_{local}(e) \tag{S35}$$

In context of disordered systems, it is believed that $\Omega_e \sim E_c$ with E_c as the Thouless energy and is of the order of few mean level spacing Δ_e at energy e.

The evolution equation for the JPDF of the components $\psi_\mu \equiv \langle \mu | \Psi \rangle$ of an arbitrary eigenfuntion Ψ of a Hamiltonian H taken from the ensemble (S1) can be given as

$$\frac{\partial P_\Psi}{\partial \Lambda} = \left[\sum_{m,n} \frac{\partial^2}{\partial \psi_\mu \partial \psi_\nu} h_2 + \sum_n \frac{\partial}{\partial \psi_\nu} h_1 \right] \tag{S36}$$

where, $h_1 \equiv (N-1)\psi_\nu P_\Psi$ and $h_2 \equiv (\delta_{\mu\nu} - \psi_\mu \, \psi_\nu) P_\Psi$ and $\Lambda(Y - Y_0, e) = \frac{Y - Y_0}{\Omega_e^2}$, later referred as the *strength* complexity parameter. It is worth noting its difference from the *spectral complexity parameter* Λ_e that appears in the spectral statistcs [1,2]: $\Lambda_e(Y - Y_0, e) = \frac{Y - Y_0}{\Delta^2_{local}}$.

Due to latter appearing in eigenfunction statistics and

Equation (S27) is derived from Equation (S22) without any approximation but the derivation of Equation (S22) described in previous section is based on second order perturbation theory and Markovian approximation. However these approximations are not really needed for a Gaussian $\rho(H)$ and, as discussed in [3], Equation (S27) can be derived from an exact route i.e an exact diagonalization of the evolution equation (S2) for $\rho(H)$. (We note that the notation P_Ψ was replaced, in [3], by P_{N1} and ψ_m by ψ_{mk}, see Equation (18) of [3]).

4. Derivation of Equation (S34)

Consider the integral

$$Q^{rs}_{mn;k} = \sum_{j=1;j\neq k}^{N} \int \frac{(O_{mj}O_{nj})^r}{(e_k-e_j)^s} P_{N2}\, \mathrm{d}e_j \mathrm{D}\psi_j, \tag{S37}$$

The correlation between eigenvalues in random matrix ensembles are known to decay rapidly with their separation, with those beyond a few mean level spacings are uncorrelated. For distances $|e_k - e_j| > N_k\Delta_e$, with $N_k \sim O(1)$, one can then approximate $P_2(O_k, O_j, e_k, e_j) \approx P_1(O_k, e_k)\; P_1(O_j, e_j)$. The above integral can now be rewritten as

$$Q^{rs}_{mn;k} = (E_1 + E_2 + E_3) \tag{S38}$$

$$E_1 = \sum_{j=1;j\neq k}^{N} \int_{-\infty}^{e_k-\Omega_k/2} \frac{(O_{mj}O_{nj})^r}{(e_k-e_j)^s}\; P_1(O_k,e_k)\; P_1(O_j,e_j)\; \mathrm{d}e_j \mathrm{D}O_j, \tag{S39}$$

$$E_2 = \sum_{j=1;j\neq k}^{N} \int_{e_k-\Omega_k/2}^{e_k+\Omega_k/2} \frac{(O_{mj}O_{nj})^r}{(e_k-e_j)^s}\; P_2(O_k,O_j,e_k,e_j)\; \mathrm{d}e_j \mathrm{D}O_j, \tag{S40}$$

$$E_3 = \sum_{j=1;j\neq k}^{N} \int_{e_k+\Omega_k/2}^{\infty} \frac{(O_{mj}O_{nj})^r}{(e_k-e_j)^s}\; P_1(O_k,e_k)\; P_1(O_j,e_j)\; \mathrm{d}e_j \mathrm{D}O_j, \tag{S41}$$

where Ω_k is a spectral range of the order of few mean level-spacings: $\Omega_k = N_k\; \Delta_k$ with $\Delta_k(e_k)$ as the local mean level spacing and N_k as the number of eigenvalues in this range.

Using the definition

$$\langle (O_{mj}O_{nj})^r \rangle_j = \frac{N}{R_1(e_j)} \int (O_{mj}O_{nj})^r\; P_1(O_j,e_j)\; \mathrm{D}O_j \tag{S42}$$

where the notation $\langle\rangle_j$ implies an integration over O_j-space only and $R_1(e)$ is the average level density at energy e_j given as

$$R_1(e_j) = N \int P_1(O_j,e_j)\; \mathrm{D}O_j \tag{S43}$$

with $\int_{-\infty}^{\infty} R_1(e)\; \mathrm{d}e = N$, the integrals E_1, E_3 can further be written as

$$E_1 = \frac{(-1)^s}{N}\; \mathcal{F}_r\; P_1(O_k,e_k) \int_{-\infty}^{-\Omega_k/2} \frac{R_1(e_k+y)}{y^s}\; \mathrm{d}y, \tag{S44}$$

$$E_3 = \frac{1}{N}\; \mathcal{F}_r\; P_1(O_k,e_k) \int_{\Omega_k/2}^{\infty} \frac{R_1(e_k+y)}{y^s}\; \mathrm{d}y, \tag{S45}$$

where

$$\mathcal{F}_0 \equiv \sum_{j=1;j\neq k}^{N} \langle (O_{mj}O_{nj})^0 \rangle_j = (N-1) \tag{S46}$$

and

$$\mathcal{F}_1 \equiv \sum_{j=1;j\neq k}^{N} \langle O_{mj}O_{nj} \rangle_j = \sum_{j=1}^{N} \langle O_{mj}O_{nj} \rangle - O_{mk}O_{nk}. \tag{S47}$$

https://doi.org/10.3390/e28010029

Due to confinement of the eigenvalues, R_1 decays for large spectral-ranges and main contribution to the integral in E_1 comes from the neighborhood of $y \sim -\Omega_k/2$. One can then approximate E_1 as

$$E_1 = \frac{1}{N}\frac{2^s}{\Omega_k^s}\mathcal{F}_r\, P_1(O_k, e_k)\int_{-\infty}^{-\Omega_k/2} R_1(e_k+y)\, \mathrm{d}y, \tag{S48}$$

Similarly E_3 becomes

$$E_3 = \frac{1}{N}\frac{2^s}{\Omega_k^s}\mathcal{F}_r\, P_1(O_k, e_k)\int_{\Omega_k/2}^{\infty} R_1(e_k+y)\, \mathrm{d}y, \tag{S49}$$

Now as $\int_{-\infty}^{-\Omega_k/2} R_1(e_k+y)\,\mathrm{d}y + \int_{\Omega_k/2}^{\infty} R_1(e_k+y)\,\mathrm{d}y = \int_{-\infty}^{\infty} R_1(e_k+y)\,\mathrm{d}y - \int_{-\Omega/2}^{\Omega/2} R_1(e_k+y)\,\mathrm{d}y \approx N - N_k$, we have

$$E_1 + E_3 \approx \frac{2^s}{\Omega_k^s}\mathcal{F}_r\, P_{N1}(\psi_k, e_k) \tag{S50}$$

To calculate E_2, we note that the integral over y in Equation (S51) is confined over a very small spectral range Ω_k around e_k. As the average correlation between components of an eigenfunction is expected to be almost same as another eigenfunction if their eigenvalues are approximately equal. Thus for $e_j \in \Omega_k$, one can approximate $\overline{\langle O_{nj}O_{mj}\rangle}_{e_k} \approx \overline{\langle O_{nk}O_{mk}\rangle}$ where $\overline{\langle O_{nj}O_{mj}\rangle}_{e_k}$ is the ensemble as well as spectral averaged local correlation of an eigenstate with its energy close to e_k: $\overline{\langle O_{nj}O_{mj}\rangle}_{e_k} = \frac{1}{\Omega_k}\int_{\Omega_k}\langle O_{nj}O_{mj}\rangle\,\mathrm{d}e$. This leads to

$$E_2 \approx (-1)^s\,(N-1)\,\overline{\langle (O_{nk}O_{mk})^r\rangle}\int_{-\Omega_k/2}^{\Omega_k/2} y^{-s}\,\mathcal{P}_2(O_k, e_k, y)\,\mathrm{d}y \tag{S51}$$

where $\mathcal{P}_2(O_k, e_k, y) = \int P_2(O_k, O_j, e_k, y)\,\mathrm{D}O_j$. Now expanding $\mathcal{P}_2(\psi_k, e_k, e_k+y)$ in Taylor's series around $y = 0$, Equation (S51) can be approximated as

$$E_2 \approx (-1)^s\,(N-1)\,\overline{\langle (O_{nk}O_{mk})^r\rangle}\sum_{n=0}^{\infty}\frac{\alpha_n}{n!}\frac{\mathrm{d}^n\mathcal{P}_{N2}}{\mathrm{d}y^n}\Big|_{y=0} \tag{S52}$$

where $\alpha_n = \int_{-\Omega_k/2}^{\Omega_k/2} y^{n-s}\,\mathrm{d}y$. Neglecting terms with higher powers of Ω_k, the above leads to, for $s = 2$, $E_2 \approx \alpha_0\,\mathcal{P}_2(O_k, e_k, 0) = -\frac{4}{\Omega_k}\,\mathcal{P}_2(O_k, e_k, 0)$.

Following the definition $P_1(O_k, e_k) = \int_{-\infty}^{\infty}\mathcal{P}_2(O_k, e_k, e_k+y)\,\mathrm{d}y$, one can write $\mathcal{P}_2(O_k, e_k, 0) \propto P_1(O_k, e_k)$. As $\Omega_k \sim E_c \ll 1$, the contribution from E_2 is negligible as compared to E_1, E_3. Substitution of Equation (S50) in Equation (S38) now leads to

$$Q_{mn;k}^{rs} \approx \left(\frac{2}{\Omega_k}\right)^s \mathcal{F}_r\, P_1(O_k, e_k) \tag{S53}$$

where $\mathcal{F}_0$ and $\mathcal{F}_1$ are given by Equation (S46) and Equation (S47). Further noting that the contribution from the terms with different j in the sum fluctuate rapidly between positive and negative values, the $\sum$ over $j = 1 \to N$ is expected to be non-zero only if $m = n$. Thus using the approximation $\sum_{j=1;j\neq k}^{N}\langle O_{mj}O_{nj}\rangle_j = \delta_{mn}$, we further have

$$\mathcal{F}_1 \approx \delta_{mn} - O_{mk}O_{nk}. \tag{S54}$$

5. Single-particle entanglement dynamics: derivation of equations given in section V of main text

An eigenstate of the single-particle Anderson Hamiltonian can be written in the site basis as,

$$|\psi\rangle = \sum_{r \in A \cup B} \psi_r |1\rangle_r \otimes_{r' \neq r} |0\rangle_{r'}, \tag{S55}$$

where, A and B are the two bi-partitions, created, for example, slicing the 3D lattice horizontally. The above state can then be written as a superposition of states where the particle occupies either the subsystem A or B:

$$|\psi\rangle = |1\rangle_A |0\rangle_B + |0\rangle_A + |1\rangle_B, \tag{S56}$$

where, $|1\rangle_A = \sum_{r \in A} \psi_r |1\rangle \otimes_{r' \neq r} |0\rangle_{r'}$ and $|0\rangle_A = \otimes_{r \in A} |0\rangle_r$; similarly, for B. We define,

$$P_A \equiv \langle 1|1\rangle_A = \sum_{r \in A} |\psi_r|^2, \tag{S57}$$

5.1. Λ-dependence of $\langle P_A \rangle$: derivation of Equation (40) of the main text

To derive the Λ-governed evolution equation for P_A, we proceed as follows. The average of P_A over the eigenfunction ensemble represented by P_ψ can be defined as

$$\langle P_A \rangle = \int_{-\infty}^{\infty} P_A \, P_\psi \, D\psi, \tag{S58}$$

A differentiation of both the sides with respect to Λ, we have,

$$\frac{\partial \langle P_A \rangle}{\partial \Lambda} = \int_{-\infty}^{\infty} P_A \, \frac{\partial P_\psi}{\partial \Lambda} \, D\psi, \tag{S59}$$

where, $D\psi \equiv \prod_i d\psi_i$. Substituting Equation (S27) on the right side of the above equation and using the definition in Equation (S57), we get,

$$\frac{\partial \langle P_A \rangle}{\partial \Lambda} = \sum_{m,n} \int_{-\infty}^{\infty} P_A \left(\frac{\partial^2 h_2}{\partial \psi_n \partial \psi_m} \right) P_\psi \, D\psi + \sum_n \int_{-\infty}^{\infty} P_A \left(\frac{\partial h_1}{\partial \psi_n} \right) P_\psi \, D\psi \tag{S60}$$

$$= \int_{-\infty}^{\infty} \sum_{n \in A} 2(1 - \psi_n^2) \, P_\psi \, D\psi - 2(N-1) \int_{-\infty}^{\infty} \sum_{n \in A} \psi_n^2 \, P_\psi \, D\psi \tag{S61}$$

where, the second equation is obtained by using repeated partial integration and by noting that P_ψ vanishes at $\psi_i \to \pm\infty \ \forall i$. Rearranging the terms on the right side of the above equation and using the definition (S57) now leads to

$$\frac{\partial \langle P_A \rangle}{\partial \Lambda} = 2 \sum_{n \in A} \int_{-\infty}^{\infty} P_\psi \, D\psi - 2N \int_{-\infty}^{\infty} P_A \, P_\psi \, D\psi \tag{S62}$$

$$= 2(N_A - N\langle P_A \rangle). \tag{S63}$$

Here the second relation follows by invoking the normalization condition $\int_{-\infty}^{\infty} P_\psi \, D\psi = 1$. As a check, we note that, for the balanced bi-partition $N = 2N_A$, the above equation gives correct limiting behavior as $\Lambda \to \infty$: $\langle P_A \rangle \to 1/2$. Further, for solving Equation (S63), we need to choose the initial condition carefully. $\Lambda \to 0$ correspond to the localized state. The localization center can, however, belong to either region A or B, depending on which, P_A is either 0 or 1. The presence or absence of the localization center in region A is a completely random event.

For analyzing the statistics of P_A, in this work, for a fixed set of parameters, we filter eigenstates from all the samples (set of states near $E = 0$ for a disorder realization and for many such disorder realizations) whose localization center lie in A; such that we have, $\langle P_A \rangle(\Lambda = 0) = 1$. The solution of Equation (S63) can now be given as

$$\langle P_A(\Lambda) \rangle = \frac{1}{2}\left[1 + e^{-4N_A\Lambda}\right]. \tag{S64}$$

5.2. Dynamics of Single Particle Entanglement Entropy S_A

5.2.1. Λ**-dependence of the average** $\langle S_A \rangle$**: derivation of Equation (34) of the main text**

We have,

$$S_A(\rho_A) = -P_A \log P_A - P_B \log P_B, \tag{S65}$$

where, $P_B \equiv 1 - P_A$. For deriving the evolution equation for $\langle S_A \rangle$, we first note the following are true,

$$\frac{\partial S_A}{\partial \psi_n} = \begin{cases} -2\psi_n \log P_A - 2\psi_n, \; n \in A \\ -2\psi_n \log P_B - 2\psi_n, \; n \in B \end{cases} \tag{S66}$$

and,

$$\frac{\partial^2 S_A}{\partial \psi_m \psi_n} = \begin{cases} -2(1 + \log P_A)\delta_{mn} - \frac{4\psi_n\psi_m}{P_A}, \; m,n \in A \\ -2(1 + \log P_B)\delta_{mn} - \frac{4\psi_n\psi_m}{P_B}, \; m,n \in B. \end{cases} \tag{S67}$$

As before, the evolution of $\langle S_A \rangle$ is defined as,

$$\frac{\partial \langle S_A \rangle}{\partial \Lambda} = \int S_A \frac{\partial P_\psi}{\partial \Lambda}\, D\psi. \tag{S68}$$

Substituting the Equation (S27) into the above equation and using Eqs. (S66) and (S67), we get,

$$\begin{aligned}
\frac{\partial \langle S_A \rangle}{\partial \Lambda} &= -\sum_{m,n\in A} \int \left[2(1 + \log P_A)\delta_{mn} + 4\frac{4\psi_m\psi_n}{P_A}\right](\delta_{mn} - \psi_n\psi_m)\; P_\psi\; D\psi, \\
&\quad -\sum_{m,n\in B} \int \left[2(1 + \log P_B)\delta_{mn} + 4\frac{4\psi_m\psi_n}{P_B}\right](\delta_{mn} - \psi_n\psi_m)\; P_\psi\; D\psi, \\
&\quad +\sum_{n\in A} \int_z 2\psi_n(1 + \log P_A)(N-1)\psi_n\; P_\psi\; D\psi + \sum_{n\in B} \int 2\psi_n(1 + \log P_B)(N-1)\psi_n\; P_\psi\; D\psi, \\
&= -\sum_{n\in A} \int \left[2(1 + \log P_A) + \frac{4\psi_n^2}{P_A}\right](1 - \psi_n^2)\; P_\psi\; D\psi - \sum_{(m\neq n)\in A} \int \frac{4\psi_m^2\psi_n^2}{P_A}\; P_\psi\; D\psi. \\
&\quad -\sum_{n\in B} \int \left[2(1 + \log P_B) + \frac{4\psi_n^2}{P_B}\right](1 - \psi_n^2)\; P_\psi\; D\psi - \sum_{(m\neq n)\in B} \int \frac{4\psi_m^2\psi_n^2}{P_B}\; P_\psi\; D\psi. \\
&\quad +2(N-1) \int (P_A \log P_A + P_B \log P_B + P_A + P_B)\; P_\psi\; D\psi \\
&= 2(N-1) - 2(N-1)\langle S_A \rangle - \int (2N + 2N_A \log P_A + 2N_B \log P_B + 2S_A + 2)\; P_\psi\; D\psi.
\end{aligned} \tag{S69}$$

Simplifying the above equation, we get

$$\frac{\partial \langle S_A \rangle}{\partial \Lambda} = -4 - 2N\langle S_A \rangle - 2N_A\langle \log P_A \rangle - 2N_B\langle \log P_B \rangle. \tag{S70}$$

For the balanced case, $N_A = N_B = \frac{N}{2}$, we have

$$\frac{\partial\langle S_A\rangle}{\partial\Lambda} = -4 - 2N\langle S_A\rangle - N\langle\log P_A P_B\rangle. \tag{S71}$$

5.2.2. **Λ-dependence of the variance $\langle(\delta S_A)^2\rangle$: derivation of Equation (45) of the main text**

Since, the variance $\langle(\delta S_A)^2\rangle \equiv \langle S_A^2\rangle - \langle S_A\rangle^2$, we begin by calculating $\langle S_A^2\rangle$. First, we note that,

$$\frac{\partial(S_A^2)}{\partial\psi_n} = 2S_A\frac{\partial S_A}{\partial\psi_n} \tag{S72}$$

$$\frac{\partial^2(S_A^2)}{\partial\psi_m\partial\psi_n} = 2\frac{\partial S_A}{\partial\psi_m}\frac{\partial S_A}{\partial\psi_n} + 2S_A\frac{\partial^2 S_A}{\partial\psi_m\partial\psi_n}, \tag{S73}$$

where, the terms on the right hand side of the above equations are defined in Equations (S66), and (S67). Following similar steps as before, we get,

$$\begin{aligned} -\sum_n\int\frac{\partial S_A^2}{\partial\psi_n}(N-1)\psi_n\, P_\psi\, D\psi &= \int_z 2S_A(N-1)\psi_n\sum_{n\in A}(2\psi_n\log P_A + 2\psi_n)\, P_\psi\, D\psi \\ &\quad + \int 2S_A(N-1)\psi_n\sum_{n\in B}(2\psi_n\log P_B + 2\psi_n)\, P_\psi\, D\psi \\ &= -4(N-1)\langle S_A^2\rangle + 4(N-1)\langle S_A\rangle, \end{aligned} \tag{S74}$$

and

$$\sum_{m,n}\int_z\frac{\partial^2(S_A^2)}{\partial\psi_n\partial\psi_m}(\delta_{mn}-\psi_n\psi_m)\, P_\psi\, D\psi = -(4N-12)\langle S_A\rangle - 4\Big\langle N_A S_A\log P_A + N_B S_A\log P_B\Big\rangle$$
$$-4\langle S_A^2\rangle - 8\langle S_A^2\rangle + 8\langle S_A\rangle + 8\Big\langle P_A(\log P_A)^2 + P_B(\log P_B)^2\Big\rangle. \tag{S75}$$

Using the above equations and Equation (S27), and further simplifying for large N and balanced bipartition $N_A = N_B = \frac{N}{2}$, we get

$$\begin{aligned} \frac{\partial\langle S_A^2\rangle}{\partial\Lambda} &\approx -4N\langle S_A^2\rangle + 4N_A\Big\langle\log P_A\log P_B\Big\rangle + 4N_A\Big\langle P_A(\log P_A)^2 + P_B(\log P_B)^2\Big\rangle \\ &= -4N\langle S_A^2\rangle - 4N_A\langle S_A\log(P_A P_B)\rangle. \end{aligned} \tag{S76}$$

Since,

$$\frac{\partial\langle(\delta S_A)^2\rangle}{\partial\Lambda} = \frac{\partial\langle S_A^2\rangle}{\partial\Lambda} - 2\langle S_A\rangle\frac{\partial\langle S_A\rangle}{\partial\Lambda}, \tag{S77}$$

using Equations (S70) and (S76) in the above equation, we have,

$$\frac{\partial\langle(\delta S_A)^2\rangle}{\partial\Lambda} = -4N\langle(\delta S_A)^2\rangle - 2N\,\mathrm{cov}(S_A, \log(P_A P_B)), \tag{S78}$$

where, the covariance $\mathrm{cov}(S_A, \log(P_A P_B)) \equiv \big[\langle S_A\log(P_A P_B)\rangle - \langle S_A\rangle\langle\log(P_A P_B)\rangle\big]$.

6. Calculation of complexity constants for Anderson and Rosenzweig-Porter ensemble

The condition $\frac{\partial \rho}{\partial t_\alpha} = 0$ in Equation (S5) for $\alpha > 1$, implies t_α as the constants of evolutions; the latter can be determined by solving the characteristic equation (for the case $b_{kl} \neq 0$)

$$\frac{dv_{kk}}{2(1-\gamma\, v_{kl})} = \ldots = \frac{dv_{kl}}{2(1-\gamma\, v_{kl})} = \frac{db_{kl}}{\gamma b_{kl}} = \frac{dY_\alpha}{0}. \tag{S79}$$

A general solution of the above equation is

$$F(Y_\alpha, S_{1\alpha}, \ldots, S_{M-1,\alpha}) = 0 \tag{S80}$$

where $S_{n\alpha}$ are the constant surfaces obtained by solving the above set of M differentials.

To obtain the solution of the set of equations in Equation (S79), we consider an arbitrary pair of differentials of variances, say,

$$\frac{dv_{mn}}{1-\gamma\, v_{mn}} = \frac{dv_{ij}}{1-\gamma\, v_{ij}} \tag{S81}$$

The solution of the above differential equations can now be given as

$$\log\left(\frac{1-\gamma\, v_{mn}}{1-\gamma\, v_{ij}}\right) = c_{v;mnij}, \tag{S82}$$

with $c_{v;mnij}$ as a constant.

Similarly the pairs of differentials can also be chosen from the non-zero mean values,

$$\frac{db_{mn}}{b_{mn}} = \frac{db_{ij}}{b_{ij}} \tag{S83}$$

The solution of the above differential equation can be given as

$$\log\left(\frac{b_{mn}}{b_{ij}}\right) = c_{b;mnij}, \tag{S84}$$

with $c_{b;mnij}$ as a constant. The constant so obtained can then be chosen as one of the t_α's for $\alpha > 1$. Choosing different pairs of m, n and i, j, many such constants can be defined.

Equation (S82) and Equation (S84) define a set of conditions on the ensemble parameters which can be used to determine complexity constants. The next question is whether the ensemble parameters in case of the Anderson and RP ensemble satisfy the above conditions?

Indeed, as in RP case, $v_{mn} = v_{ij} = \frac{1}{(1+\mu)}$, the above condition can easily be satisfied by any two pairs of indices m, n and i, j and we have $t_\alpha = \left(\frac{1-\gamma\, v_{mn}}{1-\gamma\, v_{ij}}\right) = 1$ for all $\alpha > 1$.

Similarly, for Anderson case, with same variances for the hopping off-diagonals, the above condition can be satisfied by a site represented by the indices m, n and its nearest neighbor site by indices $m_d n_d$. We can now choose $t_\alpha = \left(\frac{1-\gamma\, v_{mn}}{1-\gamma\, v_{m_d n_d}}\right) = 1$ for all $\alpha > 1$. As in this case the mean values of some of the matrix elements are non-zero, more constants can be defined by their consideration.

We note that the existence of a solution of the type mentioned above is based on exploiting the relations among different variances and mean values (e.g. $v_{mn} \propto v_{ij}$ satisfied by all pairs in RP case and for specific pairs i.e. those representing nearest neighbor

sites of Anderson case) and need not exist for all ensembles. The alternative solutions leading to constants t_α can then be obtained, e.g., by considering combinations of more than a pair of differentials (e.g $dY_\alpha \equiv \sum_{mn} \frac{q_{mn;\alpha}\, dv_{mn}}{1-\gamma v_{mn}} = 0$ with $\sum_{mn} q_{mn;\alpha} = 0$ with $q_{mn;\alpha}$ arbitrary functions). Indeed as indicated by the study [4,5], a matrix of $N \times N$ has many basis constants which can be chosen as the constants of evolution t_α; this implies that the transformation $v, b \to Y$-space can always be defined if the basis used for the dynamics is kept fixed.

7. Implications of complexity parameters based formulations [3]

Equation (S27) describes a common mathematical formulation for a typical eigenfunction of the Hamiltonians modeled by Equation (S1); the information about the system enters in the formulation through the *strength complexity parameter* $\Lambda(e) = \frac{Y-Y_0}{\Omega_e^2}$ as well system size N. The explicit appearance of the latter (besides implicitly through Λ) in Equation (S27) suggests a lack of finite size scaling in the distributions of the eigenfunction fluctuations and an absence of their critical limit. This is in contrast with the spectral statistics which is sensitive to *spectral complexity parameter* $\Lambda_e(e) = \frac{Y-Y_0}{\Delta_{local}^2}$ and shows a single-parametric scaling as well as a critical behavior if following condition is satisfied

$$\Lambda_e^* \equiv \lim_{N\to\infty} \Lambda_e = \text{finite} \tag{S85}$$

An important point worth noting here is the relation between the parameters Λ and Λ_e governing the statistics of the eigenfunctions and eigenvalues, respectively: $\Lambda = \chi\, \Lambda_e$ where

$$\chi = \frac{\Delta_{local}^2}{\Omega_e^2} \sim \frac{1}{N^2\, \langle I_2(e)\rangle^2} \tag{S86}$$

The relation is useful by noting the important role of Λ_e in locating the critical point of the spectral statistics. As both $|Y - Y_0|$ as well as R_{local} are functions of N, the latter can affect Λ_e significantly and indeed plays a crucial role in determining the critical spectral statistics. For finite systems, the statistics smoothly approaches one of the two end points, namely, $\Lambda_e \to 0$ or $\Lambda_e \to \infty$, with increasing system size. The variation of Λ_e in infinite systems, however, may lead to an abrupt transition of the statistics, with its critical point given by the condition $\Lambda_e =$ *size independent*. The finite, non-zero Λ_e strength, say Λ_e^*, at the critical point, results in a spectral statistics different from the two end points. Note, however, that the existence of a critical point or its absence depends on the relative size-dependencies of $|Y - Y_0|$ and the local mean level spacing Δ_{local}. If the size-dependence of Δ_{local}^2 remains different from that of $|Y - Y_0|$ under all complexity conditions, Λ_e would never achieve a finite non-zero value in infinite size limit. As a consequence, such a system is not expected to support a critical behavior of spectral statistics.

While Λ_e becomes size-independent at the critical point of the spectral statistics, the eigenfunction statistics governed by $\Lambda = \chi\Lambda_e$ however remains sensitive to size N (due to N appearing in right side of Equation(S27)). This in turn results in a multifractal behavior of the eigenfunctions at the critical point, with scaling exponents, referred as critical exponents or multifractal dimensions, dependent on system parameters [3,6]. As mentioned above, changing system parameters, in finite size systems, can change Λ_e continuously between 0 and ∞ which may lead to intermediate stages of varying degree of multifractality [6]. The physically interesting cases however usually correspond to infinite sizes where Λ_e takes only three possible values, namely, $\Lambda_e = 0, \infty, \Lambda_e^*$; for these cases therefore only one multifractal stage, that is at the critical point corresponding to Λ_e^*, can exist. As Λ_e^* is sensitive to system-specifics, the critical (multifractal) exponents can vary from system to

system. Note, as already mentioned above, the occurrence of a critical point and, therefore, a multifractal behavior of eigenstates is not a necessary feature of all infinite size complex systems.

The parameter Λ, being a function of the distribution parameters of the matrix elements, is sensitive to changes in the system parameters; this is due to latter's influence on the uncertainties associated with system-interactions. Some examples of such system parameters are disorder, dimensionality, boundary and topological conditions, system size etc. For example, the presence of disorder randomizes the interactions, with degree of disorder affecting the distribution parameters h, b and consequently Λ. The dependence of Λ on the dimensionality and boundary conditions originates from their influence on the basis connectivity i.e. degree of sparsity of the matrix which is reflected in the distribution parameters v, b. For example, for nearest neighbor hopping and hard wall boundary conditions in d-dimensions, the matrix element $H_{jk} \neq 0$ only if $j = |k \pm L^{d-1}|$ (with L as linear size). The variance v_{jk} of the distribution $\rho(H_{jk})$ is therefore finite only for $j = k$ or $|j = k \pm L^{d-1}|$ and is zero for all other j, k. The information about dimensionality in Λ also enters through the local mean level spacing which depends on the correlation volume ξ^d. (See also [2] where the dependence of Λ on system parameters is explained by considering an example of Anderson Hamiltonian).

As discussed in detail in [3], the Λ-governed evolution equation (S27) can further be used to derive the evolution of the probability density of any other measure, say $X = F(\psi)$, that can be described in terms of the eigenfunction components of a single eigenfunction, defined as $P_x(X) = \int \delta(X - F(\psi))\, P_\psi\, \mathrm{D}\psi$. The parameter, say Λ_x governing the evolution is however measure-specific; this is In contrast to spectral fluctuation with all related measures governed by Λ_e only. The solution $P(X, \Lambda_x|X_0, 0)$ of the related evolution equation describes the probability of the measure, say X, at Λ_x for a given initial state of $X = X_0$. Here P_x is subjected to an initial constraint $\lim■ \to 0 P(X, \Lambda_x|X_0, 0) = \delta(X - X_0)$. By integration of the solution over the distribution of initial values $P_0(X_0, 0)$, one can recover $P(X, \Lambda_x)$, that is, the distribution of measure X for a system with complexity parameter strength Λ:

$$P_x(X, \Lambda_x) = \int P(X, \Lambda_x|X_0, 0) P_0(X_0, 0) \mathrm{d}X_0. \tag{S87}$$

As explained in [3], through analysis of some eigenfunction fluctuation measures e.g. average inverse participation ratio etc., Λ_x is in general related to Λ_e. The energy-dependence of the latter along with existence of nonzero correlations between eigenfunctions and eigenvalues suggest the multifractality measures to be sensitive to chosen energy regime.

Equation (S87) implies that the statistics evolved in "time" Λ_x is sensitive to the collective behavior of system parameters contributing to Λ_x and the initial distribution only. The latter can always be chosen same for the systems operating in the matrix spaces of subjected to similar symmetry conditions and conservation laws; (the initial values of their Y parameters need not be equal). This can further be explained as follows: consider two different physical systems labelled as "S1" and "S2", both represented by the ensemble (S1), their different system conditions giving rise to different sets of ensemble parameters labelled as $''x1''$ and $''x2''$. If the physical conditions in "S1" and "S2" are such that the condition $\Lambda_{S1}(e_1) = \Lambda_{S2}(e_2) \equiv \Lambda$ is fulfilled, the statistical behaviour of the systems "S1" and "S2", at energy e_1, e_2 respectively, is predicted to be analogous. The above analogy however does not imply the analogy for previous histories of the two systems (indeed they may show completely different behavior for $0 < \Lambda_{x1}, \Lambda_{x2} < \Lambda_x$), their future or even other energies. For example, consider the cases of a three dimensional disordered system, say A, and a clean, closed quantum dot, say B. In the first case, $\Lambda_{x,A} = \Lambda_{x,disorder}$ is a

function of disorder, hopping strength, dimensionality, boundary condition etc. In the case of a dot, $\Lambda_{x,B} = \Lambda_{x,dot}$ is a function of shape as well as size. It is well-known that, in strong disorder limit and for circular shape, respectively, both systems show localized wavefunction dynamics and same statistical behavior of the eigenfunctions and eigenvalues of the Hamiltonians. Reducing the degree of disorder or change of shape of the dot from circle to stadium type results in a transition from localized to delocalized dynamics of the wavefunctions. The statistics in the intermediate stages during the transition for each case is governed by the respective Λ_x strengths. If, however, the $\Lambda_{x,dot} = \Lambda_{x,disorder}$ at some shape parameter and disorder strength, respectively, our analysis predicts a same statistical behavior for both systems. The implication can also be extended to classical systems e.g. stock market fluctuations which are analyzed by statistical studies of the correlation matrix of stocks [**?**]. Here a very weak interaction among certain stocks due to various socio-economic conditions results in a localized dynamics of the eigenfunctions. The changing conditions may lead to a more homogenized interaction of some of the stocks, thus introducing a transition from localized to delocalized wave dynamics. In this case, Λ is a function of the socio-economic parameters (SEP). However if $\Lambda_{x,stock} = \Lambda_{x,dot}$ for some combinations of SEP and dot parameters, respectively, the spectral and strength fluctuations in correlation matrix of the stock market and the Hamiltonian of quantum dot will show same behavior. Note the analogy of statistical behavior of the eigenvalues and eigenfunctions among the three systems, mentioned above, has already been numerically verified in delocalized waves limit $\Lambda \to \infty$). .

The above universality makes Λ formulation useful as it can be exploited to obtain the statistics of a complex system if the same information is available about another system (under same symmetry conditions) by another method. For example, for Anderson type disordered Hamiltonian, the distributions of many measures are known by non-linear sigma model techniques. The formulations can then be used for the complex systems e.g. stock markets undergoing a transition from localized $\to$ delocalized wave dynamics; one just needs to replace Λ(Anderson) by that of the system. The existence of a mathematical formulation of both eigenfunctions and eigenvalues common to a wide range of complex systems indicates the possibility of a similar formulation for many physical properties. This also suggests the possibility to classify them in various universality classes defined by the complexity parameter.

8. Local mean level density

The term "local" in local mean level density R_{local} signifies its strong dependence on the underlying eigenfunction dynamics; for the eigenfunctions with non-ergodic dynamics, it can be significantly different from the mean level density $R_1(e) = \langle \sum_n \delta(e - e_n) \rangle$. This can be explained as follows: while $R_{local}(e)$ corresponds to only those levels at energy e which are occupying the same region in basis space, $R_1(e)$ refers to all levels at energy e irrespective of their location in the basis-space. (This is because levels with eigenfunctions localized in different parts of basis space need not interact but $\Delta(e)$ is obtained by taking into account all levels in the spectrum irrespective of their interaction). Based on the above reasoning, one possible definition can be given as follows:

$$R_{1,local}(e) = \sum_n \phi_n \, \delta(e - e_n) \tag{S88}$$

with ϕ_n as the probability of nth eigenfunction occupying the same region as other eigenfunctions with energies close to e. For example, for the case in which a typical eigenfunction is delocalized in entire Hilbert space, the above implies $\phi_n = 1$ which gives $R_{ocal}(e) = R_1(e)$. (The numerical analysis for Rosenzweig-Porter ensemble indicates the

relation $R_{local}(e) = R_1(e)$ to be valid if the matrix is not sparse i.e. all off-diagonals are of the same order). Similarly, in the case of localized dynamics, although two localized states do not typically overlap but can be localized in the same region with a small probability of ξ^d/N (with $\xi(e)$ as the average localization range at spectral point e, d as the system-dimension and N as the number of basis states). This implies $\phi_n \sim \frac{\xi^d}{N}$ and thereby leads to $R_{1,local}(e) = R_1(e)\ \frac{\xi^d}{N}$ (with ξ^d/N as the probability of eigenfunctions localized in the same region of basis space). For Hermitian cases where the eigenfunctions are exponentially localized e.g. in standard Anderson Hamiltonian (a single particle moving in a random potential), ξ^d can be approximated by the average inverse participation ratio $\langle I_2 \rangle$ of the eigenfunctions with energies in the neighbourhood of the energy of interest : $\xi^d \approx (\langle I_2 \rangle)^{-1}$. The latter relation however is believed to be not valid for spectral edges (to best of my knowledge) and one may need to use alternate routes to determine ξ.

The Figure S1 displays a comparison of the inverse participation ratio I_2 for an eigenfunction at $e = 0$ for two different hopping ranges of the Anderson Hamiltonian (matrix size $N = 12^3$ and for a fixed off-diagonal disorder $w_1 = 1.0$). A variation of diagonal disorder w leads to change in $Y - Y_0$ and I_2. The label $k = 1$ and $k = 2$ correspond to nearest and next-nearest neighbour hopping, respectively. Here $s = 0.02$ for $k = 1$ and $s = 0.0$ for $k = 2$. With I_2 for $k = 1$ and $k = 2$ being analogous with changing $Y - Y_0$, Equation (S86) gives $\chi \sim 1$.

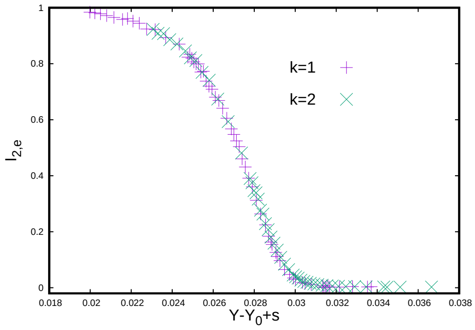

Figure S1. Comparison of inverse participation ratio I_2 for an eigenfunction at $e = 0$ for two different hopping ranges of the Anderson Hamiltonian (matrix size $N = 12^3$ and for a fixed off-diagonal disorder $w_1 = 1.0$). A variation of diagonal disorder w leads to change in $Y - Y_0$ and I_2. The label $k = 1$ and $k = 2$ correspond to nearest and next-nearest neighbour hopping, respectively. Here $s = 0.02$ for $k = 1$ and $s = 0.0$ for $k = 2$.

References

1. Shukla, P. Random matrices with correlated elements: A model for disorder with interactions. *Phys. Rev. E* **2005**, *71*, 026226. https://doi.org/10.1103/PhysRevE.71.026226.
2. Shukla, P. Level statistics of Anderson model of disordered systems: Connection to Brownian ensembles. *J. Phys.: Condens. Matter* **2005**, *17*, 1653–1677. https://doi.org/10.1088/0953-8984/17/10/020.
3. Shukla, P. Eigenfunction statistics of complex systems: A common mathematical formulation. *Phys. Rev. E* **2007**, *75*. https://doi.org/10.1103/PhysRevE.75.051113.
4. Gleit, A.; Lazar, A.J. Basis constants for the space of n× n matrices. *J. Funct. Anal.* **1976**, *22*, 354–365.
5. Megginson, R.E. *An introduction to Banach space theory*; Vol. 183, Springer Science & Business Media, 2012.
6. Sadhukhan, S.; Shukla, P. Average entropy of a subsystem. *Phys. Rev. B* **2017**, *96*, 012109.
